# Application of the Wittig Rearrangement of *N*-Butyl-2-benzyloxybenzamides to Synthesis of Phthalide Natural Products and 3-Aryl-3-benzyloxyisoindolinone Anticancer Agents

**DOI:** 10.3390/molecules29194722

**Published:** 2024-10-06

**Authors:** R. Alan Aitken, Francesca K. Cooper, Andrew D. Harper, Ryan A. Inwood, Elizabeth A. Saab, Ewan J. Soutar

**Affiliations:** EaStCHEM School of Chemistry, University of St Andrews, North Haugh, St. Andrews KY16 9ST, Fife, UK

**Keywords:** [1,2]-Wittig rearrangement, 3-arylphthalides, 3-aryl-3-hydroxyisoindolinones, natural products, anticancer agents

## Abstract

Application of the [1,2]-Wittig rearrangement and cyclisation approach to 3-arylphthalides has been evaluated for the synthesis of three bioactive natural products. While this is successful in the case of crycolide, providing the second synthesis of this compound, the more sterically demanding targets isopestacin and cryphonectric acid prove not to be amenable to this approach, with the 2,6-disubstituted aryl groups causing the failure of the rearrangement and alkylation steps, respectively. Direct oxidation of the substituted benzhydrols resulting from [1,2]-Wittig rearrangement using MnO_2_ provides a new route to 3-aryl-3-hydroxyisoindolinones, and this method has been used in the synthesis of two 3-aryl-3-benzyloxyisoindolinone anticancer agents.

## 1. Introduction

**Scheme 1:**
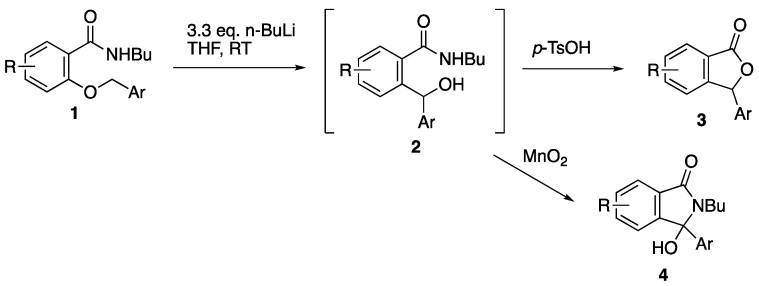
Wittig rearrangement approach to 3-arylphthalides and hydroxyisoindolinones.

In a recent paper [1], we reported that the *N*-butylamide group, C(=O)NHBu, was successful in promoting the [1,2]-Wittig rearrangement of substituted aryl benzyl ethers. While the diarylmethanol products resulting from rearrangement of 3- or 4-benzyloxy-*N*-butylbenzamides were stable, the products **2** derived from 2-benzyloxy-*N*-butylbenzamides **1** were found to cyclise slowly with loss of butylamine to give 3-arylphthalides **3**, a process accelerated in the presence of *p*-toluenesulfonic acid (Scheme 1). More recently, we have described a similar pattern of reactivity for the isomeric benzyloxyphenyl phosphonamidates where the directing group, P=O(NHBu)OEt, gives stable Wittig-rearranged diarylmethanols for the 3- or 4- series [2], but in the 2-position results in cyclisation without rearrangement to give 1,3-benzoxaphospholanes [3].

There has been considerable recent interest in the synthesis and biological activity of 3-arylphthalide natural products, and a recent review lists over 40 such products, as well as describing in detail the methods developed for their synthesis [4]. The related 3-hydroxy-3-arylisoindolinones **4** are also of considerable interest [5], particularly since some simple derivatives have shown promising anticancer activity [6,7].

In this paper, we describe our attempts to apply the Wittig rearrangement route of Scheme 1 to the synthesis of three biologically active 3-arylphthalide natural products. In addition, we disclose for the first time a new oxidative workup of the reaction products **2** from [1,2]-Wittig rearrangement of **1**, which offers a direct route to hydroxyisoindolinones **4** and its successful application to the synthesis of two anticancer compounds.

## 2. Results and Discussion

### 2.1. Synthesis of 3-Arylphthalide Natural Products

**Figure 1:**
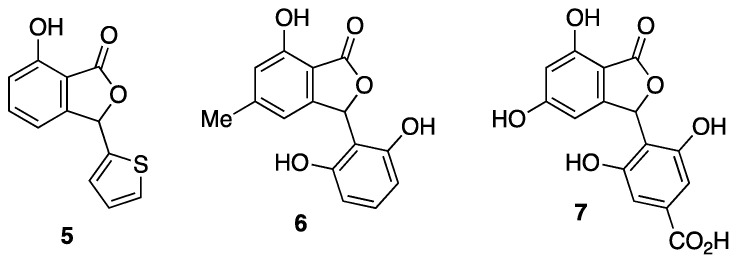
Structures of the three target phthalide natural products.

To investigate the applicability of the Wittig rearrangement–cyclisation approach to the synthesis of phthalides, we chose three examples: compounds **5**, **6** and **7** (Figure 1). Chrycolide **5** was extracted from the leaves of *Chrysanthemum coronarium* [8] and shows activity as a plant growth inhibitor. Isopestacin **6**, isolated from the endophytic fungus *Pestalotiopsis microspora* [9], has antifungal and antioxidant activity, and cryphonectric acid **7**, isolated from *Cryphonectrica parasitica* [10], showed root growth inhibition.

**Scheme 2:**
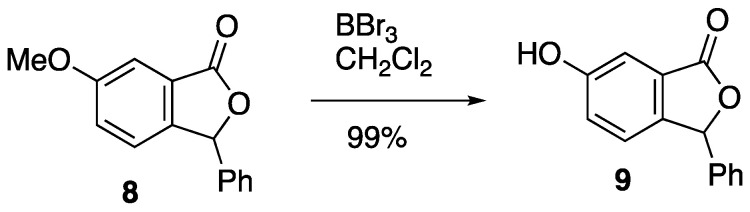
Compatibility of OMe deprotection with the presence of 3-arylphthalide.

Since our synthetic plans involved the final-stage deprotection of the phenolic OH groups carried through the syntheses as methyl ethers, we though it wise to first confirm the compatibility of the 3-arylphthalide functionality with the rather harsh conditions required for this deprotection. For this, a sample of the 5-methoxy compound **8**, already obtained by the Wittig rearrangement–cyclisation approach [1], was treated with boron tribromide in dichloromethane to afford an essentially quantitative yield of the deprotected compound **9** with no evidence of unwanted side reactions (Scheme 2).

**Scheme 3:**
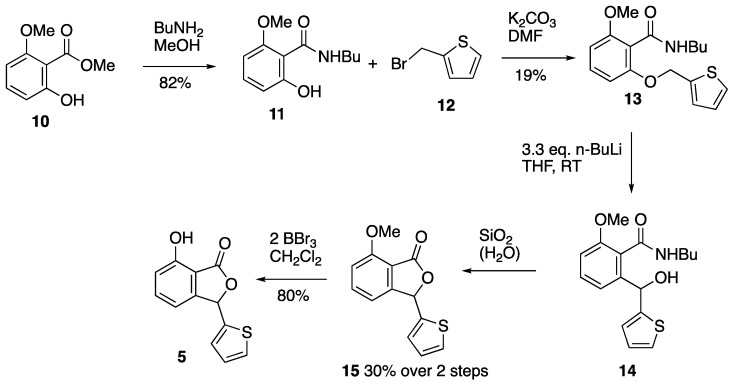
Synthetic approach to crycolide **5**.

Encouraged by this result, we embarked on the synthesis of chrycolide, as summarised in Scheme 3. The starting material, methyl 2-hydroxy-6-methoxybenzoate **10**, was readily prepared in 81% yield by mono de-methylation of methyl 2,6-dimethoxybenzoate [11] using boron trichloride and had properties in good agreement with the reported data [12]. This was converted into the *N*-butyl amide **11** by reaction with an excess of butylamine in boiling methanol. The *O*-alkylation of this with 2-bromomethylthiophene **12** and potassium carbonate in DMF afforded the target ether **13** in a disappointing 19% yield, most likely due to steric hindrance. In the key step, this was treated with 3.3 equivalents of *n*-butyllithium in THF, resulting in the Wittig rearrangement to the secondary alcohol **14**. However, this was not isolated, since it was found to cyclise spontaneously on purification by preparative TLC with the loss of butylamine to afford the phthalide **15** in low yield. The final deprotection using boron tribromide proceeded in good yield to afford chrycolide **5**, showing ^1^H and ^13^C NMR data in good agreement with those of the natural product published by Tada and Chiba (see Supplementary Materials) [8]. As far as we are aware, there is only one other published synthesis of chrycolide reported by a group at Merck in 2013 [13].

**Scheme 4:**
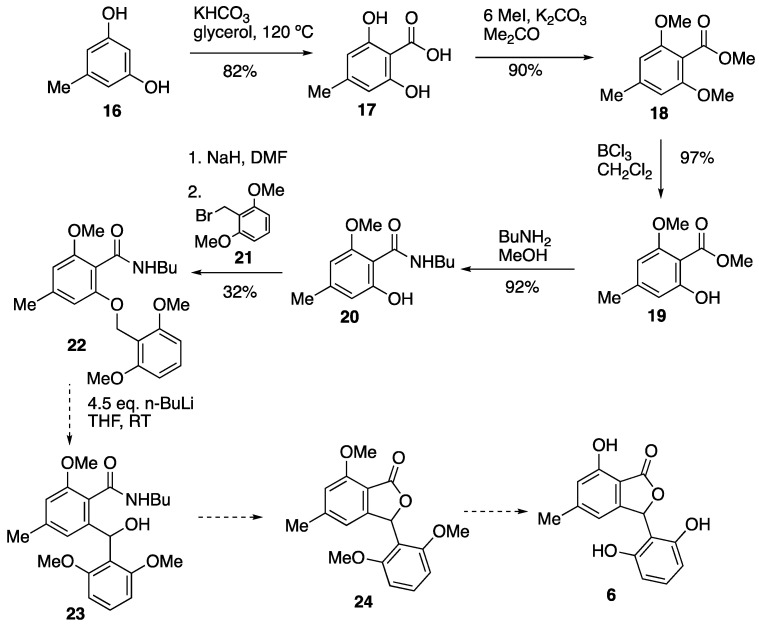
Synthetic approach to isopestacin **6**.

The structure of isopestacin contains several features making it more challenging. The ring methyl group has to be introduced at the start and, more importantly, the presence of two *ortho* substituents on the 3-aryl group may make the Wittig rearrangement step difficult. The synthetic plan, summarised in Scheme 4, starts from orcinol **16** and makes use of an unusual carboxylation [14] analogous to the Kolbe–Schmitt reaction to form the dihydroxy-*p*-toluic acid **17**. Exhaustive methylation of this gave a good yield of the dimethoxy ester **18**, and this was readily mono de-methylated in excellent yield using boron trichloride to give **19**, which had both melting point [14] and ^1^H and ^13^C NMR data [15] in agreement with reported values. Conversion into the previously unknown *N*-butyl amide **20** was achieved in high yield. The final stage in the assembly of the substrate for the key Wittig rearrangement involved alkylation with 2,6-dimethoxybenzyl bromide **21**. As we have already described elsewhere [16], this is a highly unstable compound, but by using the freshly prepared reagent immediately, the target compound **22** was obtained in reasonable yield. Unfortunately, all attempts to bring about the required Wittig rearrangement of this by treatment with *n*-butyllithium were unsuccessful. Even using 4.5 equivalents of butyllithium gave, after aqueous workup, mainly the recovered starting material accompanied by a low yield of the debenzylated product **20**. We conclude that the benzyl carbanion formed from **22** is too hindered to undergo rearrangement and is simply reprotonated upon workup. This is a little surprising, since in our previous work [1], we were able to observe successful Wittig rearrangement with a 2-methoxy-1-naphthylmethyl ether. As far as we are aware, there is only one reported synthesis for both isopestacin and cryphonectric acid [17].

**Scheme 5:**
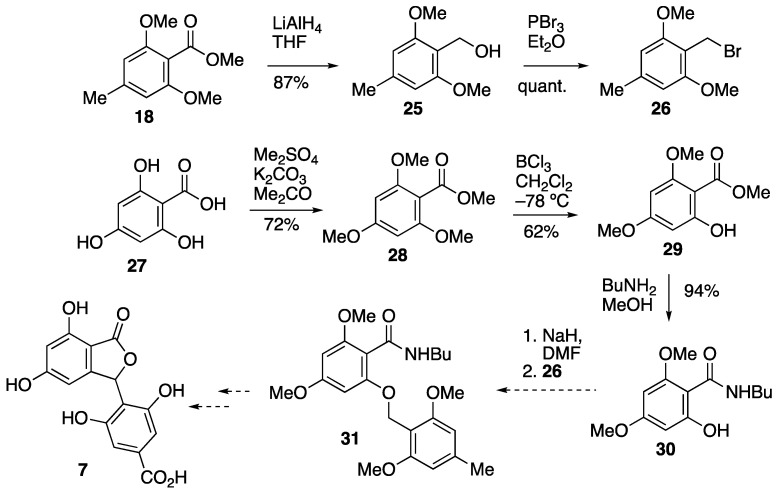
Synthetic approach to cryphonectric acid **7**.

To apply the method to the synthesis of cryphonectric acid **7**, we envisaged creating the carboxylic acid function by a late-stage oxidation of the methyl group, and so, the target for rearrangement and cyclisation became the ether **31** (Scheme 5). The required benzylic bromide **26** was not previously known, and since it was expected to be unstable like **21**, no attempt was made to isolate it. The ester **18**, already available from the previous synthesis, was subjected to lithium aluminium hydride reduction to afford **25** in good yield, and this was treated with phosphorus tribromide to generate **26** ready for immediate use.

In the meantime, the coupling partner **30** was prepared starting from trihydroxybenzoic acid **27** by exhaustive methylation to give **28**, mono de-methylation using BCl_3_ to give **29**, and reaction with *n*-butylamine in methanol to give the new compound **30**. Treatment of this with sodium hydride in DMF followed by the addition of freshly prepared **26** in DMF unfortunately gave mainly (~60%) recovered **30**, together with just a trace (2%) of the desired product **31** in an impure form. It was clear that this approach to cryphonectric acid was not going to be viable, and the synthesis was abandoned. It is notable that cyclisation approaches to phthalides bearing a hindered 3-aryl group appear to be difficult, and a recently published new route to 3-arylphthalides does not include any examples with a 2,6-disubstituted aryl group [18].

### 2.2. Synthesis of 3-Aryl-3-hydroxyisoindolin-2-ones and Their Benzyl Derivatives

**Scheme 6:**
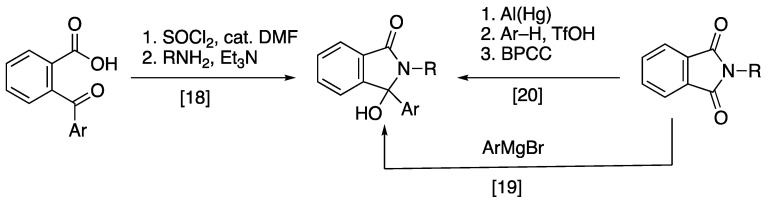
Previous routes to 3-aryl-3-hydroxy-2-alkylisoindolinones.

As mentioned in the introduction, compounds of this class have been of particular interest, since they were reported to have promising anticancer activity based on their inhibition of the MDM2-p53 protein–protein interaction [5,6]. Three main routes to the 3-hydroxy compounds required for conversion into the biologically active 3-benzyloxy derivatives have been developed (Scheme 6). The route used by Hardcastle and coworkers [5] exploits a method developed earlier by Golding and coworkers [19] and involves activation of 2-benzoylbenzoic acids with thionyl chloride followed by treatment with the requisite amine. An alternative approach developed much earlier is simply to treat the appropriate *N*-alkylphthalimide with an aryl Grignard reagent [20]. Most recently, a new method reported by Luzzio and coworker [21] involves reduction of the phthalimide, addition of the aryl group by electrophilic substitution, and a final oxidation using bipyridyl chlorochromate.

**Scheme 7:**
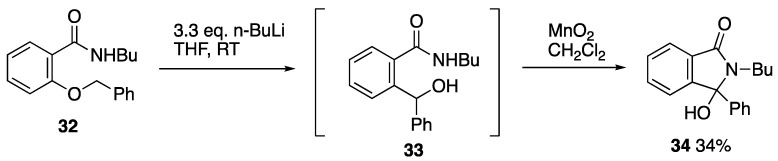
Oxidative cyclisation of the Wittig rearrangement product.

In our previous work, treatment of the 2-benzyloxy-*N*-butylbenzamide **32** with butyllithium led to Wittig rearrangement to afford the amide-substituted benzhydrol **33** [1]. This was found to be rather unstable and slowly converted with loss of butylamine into the corresponding phthalide, a process accelerated by heating in the presence of *p*-toluenesulfonic acid. We have now found that by stirring a solution of the crude product **33** in dichloromethane for 24 h with a ten-fold excess of manganese dioxide, it is possible to directly obtain the 3-hydroxy-3-phenylisoindolinone **34** in moderate overall yield (Scheme 7).

**Figure 2:**
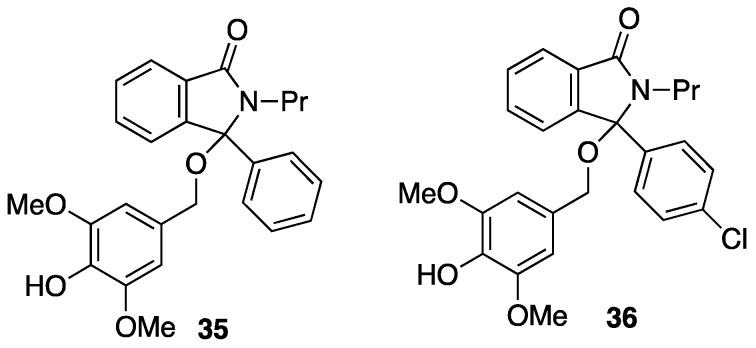
Anticancer compounds chosen as targets.

We decided to evaluate this new approach for the synthesis of some anticancer compounds and chose the two compounds **35** (IC_50_ 82 ± 8 μM) and **36** (IC_50_ 5.3 ± 0.8 μM) as targets (Figure 2). These are active as inhibitors of the MDM2-p53 protein–protein interaction [6,7]. It might be noted at this stage that the initial testing involved racemic compounds [6], as will be produced by our approach, but later studies showed that when the enantiomers of the most active compound were resolved, the activity resided predominantly in one enantiomer [7].

**Scheme 8:**
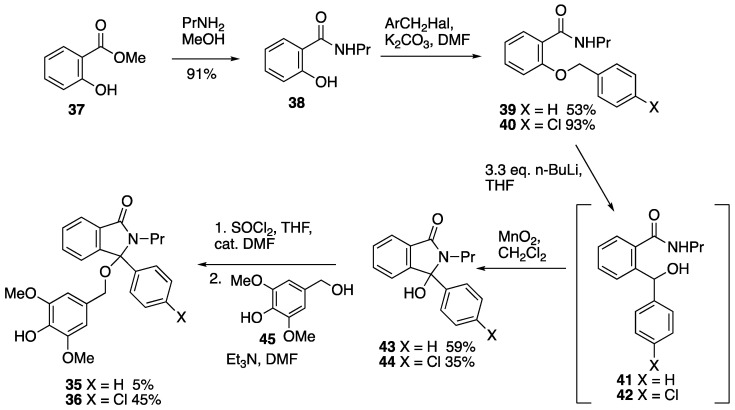
Route to anticancer compounds **35** and **36**.

The route began by converting the readily available methyl salicylate **37** into its *N*-propyl amide **38** in high yield, and this was then *O*-alkylated with either benzyl bromide to give **39** or *p*-chlorobenzyl chloride to give **40** (Scheme 8).

In the key step of the synthesis, these were then treated with 3.3 equivalents of *n*-butyllithium in THF. Since the expected products **41** and **42** were prone to cyclisation, as mentioned earlier, no attempt was made to purify these. Instead, the crude products obtained after aqueous workup, extraction, drying, and evaporation were taken up immediately in CH_2_Cl_2_ and treated with a twenty-fold excess of manganese dioxide. After filtering off the solids, evaporation, and chromatographic purification of the residue, the desired 3-hydroxyisoindolinones **43** and **44** were obtained in moderate yield.

**Figure 3:**
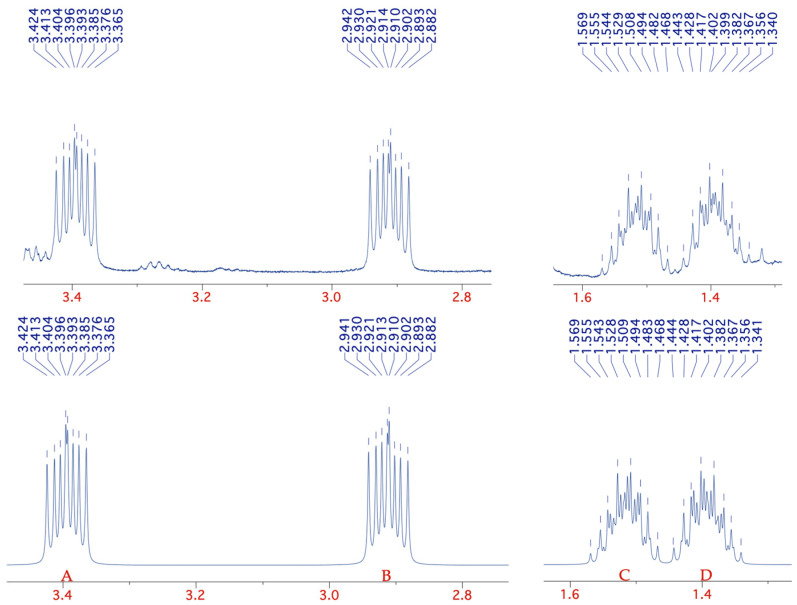
*n*-Propyl CH_2_ signals in the ^1^H NMR spectrum of **43**, (**top**, experimental spectrum and **bottom**, simulation using the values in Table 1).

The NMR spectra of these were in full agreement with previously published values [20], but the ^1^H NMR showed an interesting phenomenon: all four CH_2_ protons within the *n*-propyl groups were non-equivalent and coupled to each other resulting in a highly complex pattern (Figure 3). Although these were described in the previous work simply as multiplets, we were able to analyse them and produce a successful simulation using the values shown in Table 1 for the example of compound **43**. The same feature was apparent in the spectra of the final products **35** and **36** and is clearly a consequence of the highly hindered environment of the propyl group adjacent to the stereocentre in these compounds.

For the final stage of the synthesis, we used the reported method [6], involving activation of the hydroxyisoindolinones **43** and **44** with thionyl chloride and treatment with the commercially available 4-hydroxy-3,5-dimethoxybenzyl alcohol **45** (“syringyl alcohol”) in the presence of triethylamine. This afforded the target anticancer compounds **35** and **36** in low and moderate yields, respectively, and these had spectroscopic data identical to the published values [6].

## 3. Experimental

### 3.1. General Experimental Details

NMR spectra were recorded on solutions in CDCl_3_, unless otherwise stated, using Bruker instruments. Chemical shifts are given in ppm to high frequency from Me_4_Si, with coupling constants *J* in Hz. The ^1^H NMR spectra are referenced to internal Me_4_Si, while the ^13^C NMR spectra are referenced to the solvent signal at 77.0 (CDCl_3_). IR spectra were recorded on a Perkin Elmer 1420 instrument. Mass spectra were obtained using a Micromass instrument, and the ionisation method used is noted in each case. Preparative TLC was carried out using 1.0 mm layers of Merck alumina 60G, containing 0.5% Woelm fluorescent green indicator on glass plates. Column chromatography was carried out using silica gel (particle size 40–63 μm). Melting points were recorded on a Gallenkamp 50W melting point apparatus or a Reichert hot-stage microscope.

6-Methoxy-3-phenylisobenzofuran-1(3*H*)-one **8** was prepared, as previously described [1].

### 3.2. Trial Deprotection of a Methoxyphthalide

#### Preparation of 6-Hydroxy-3-phenylisobenzofuran-1(3*H*)-one **9**

A solution of 6-methoxy-3-phenylisobenzofuran-1(3*H*)-one **8** (0.031 g, 0.13 mmol) in CH_2_Cl_2_ (1.5 cm^3^) was stirred under nitrogen, while boron tribromide (1 M, 0.45 cm^3^, 0.42 mmol) was added. The reaction was stirred under a nitrogen atmosphere for 18 h. CH_2_Cl_2_ (10 cm^3^) and ice water (10 cm^3^) were added, and the organic layer was separated, washed with 2M hydrochloric acid (10 cm^3^), dried over magnesium sulfate and concentrated *in vacuo* to afford **9** (0.029 g, 99%); mp 201–204 °C; ^1^H NMR (300 MHz) δ_H_ 7.37 (4H, m ArCH), 7.26 (2H, m, ArCH), 7.20 (1H, d, *J* 8.5, ArCH-4), 7.15 (1H, dd, *J* 8.5, 2.5, ArCH-5), 6.36 (1H, s, 3-CH) and 5.50 (1H, br s, OH); ^13^C NMR (125 MHz) δ_C_ 170.4 (C=O), 156.8 (C–O), 142.1 (C), 136.5 (C), 129.3 (CH), 128.9 (2CH), 127.3 (C), 127.0 (2CH), 124.1 (CH), 122.8 (CH), 110.8 (CH), and 82.7 (CH); IR (ATR) ν_max_/cm^−1^ 3269 (OH), 1720 (C=O), 1321, 1308, 1065, 957 and 698; HRMS (ESI^+^) calcd. for C_14_H_11_O_3_ (M+H) 227.0708, found 227.0697.

### 3.3. Synthesis of Chrycolide ***5***

#### 3.3.1. Methyl 2-Hydroxy-6-methoxybenzoate **10**

A solution of methyl 2,6-dimethoxybenzoate [11] (3.0 g, 15.29 mmol) in CH_2_Cl_2_ (90 cm^3^) was stirred at 0 °C while boron trichloride in hexane (1M, 45 cm^3^, 45.87 mmol) was added. After the addition, the mixture was allowed to warm to RT and stirred for 18 h. The solution was poured into ice water (50 cm^3^) and a precipitate formed. The organic layer was separated, and the aqueous phase was extracted with CH_2_Cl_2_ (3 × 30 cm^3^). The combined extract was dried over magnesium sulfate, and concentrated *in vacuo* to afford **10** as a brown oil (2.25 g, 81%); ^1^H NMR (300 MHz) δ_H_ 11.52 (1H, s, OH), 7.32 (1H, t, *J* 8.4, ArC(4)H), 6.62 (1H, dd, *J* 8.4, 1.0, ArC(5)H), 6.43 (1H, dd, *J* 8.4, 0.8, ArC(3)H), 3.96 (3H, s, OCH_3_) and 3.88 (3H, s, OCH_3_). ^1^H NMR spectral data were in accordance with those previously reported [12].

#### 3.3.2. *N*-Butyl-2-hydroxy-6-methoxybenzamide **11**

A solution of methyl 2-hydroxy-6-methoxybenzoate **10** (2.00 g, 10.98 mmol) and *n*-butylamine (5.69 cm^3^, 57.6 mmol) in methanol (25 cm^3^) was heated under reflux for 18 h. Methanol was evaporated, and 2 M hydrochloric acid was added until the pH was 1. The product was extracted with CH_2_Cl_2_ (20 cm^3^). The organic layer was washed with water (3 × 20 cm^3^), dried over magnesium sulfate, and concentrated *in vacuo* to afford **11** (2.00 g, 82%); mp 165–167 °C; ^1^H NMR (300 MHz) δ_H_ 8.34 (1H, br s, NH), 7.25 (1H, t, *J* 8.4, 4-CH), 6.62 (1H, dd, *J* 8.4, 0.9, ArCH), 6.39 (1H, *J* 8.4, 0.9, ArCH), 3.93 (3H, s, OCH_3_), 3.43 (2H, m, NC*H*_2_), 1.61 (2H, m, NCH_2_C*H*_2_), 1.42 (2H, m, CH_2_C*H*_2_CH_3_) and 0.97 (3H, t, *J* 7.2, CH_2_C*H*_3_); ^13^C NMR (125 MHz) δ_C_ 170.0 (C=O), 164.3 (C–O), 158.5 (C–O), 133.0 (CH), 111.7 (CH), 103.9 (C), 100.8 (CH), 56.2 (OCH_3_), 39.0 (N*C*H_2_), 31.3 (CH_2_*C*H_2_CH_2_), 20.2 (CH_2_*C*H_2_CH_3_) and 13.7 (CH_2_*C*H_3_); IR (ATR) ν_max_/cm^−1^ 3387 (NH), 1636 (C=O), 1587, 1541, 1437, 1238, 1086, 760 and 640; HRMS (ESI^+^) calcd. for C_12_H_18_NO_3_ (M+H) 224.1287, found 224.1278.

#### 3.3.3. *N*-Butyl-2-methoxy-6-(2-thienylmethoxy)benzamide **13**

A solution of *N*-butyl-2-hydroxy-6-methoxybenzamide **11** (1.88 g, 8.42 mmol), 2-(bromomethyl)thiophene **12** (1.49 g, 8.42 mmol) and potassium carbonate (3.49 g, 6.67 mmol) in DMF (9 cm^3^) was stirred at 100 °C for 18 h. The solution was cooled to room temperature, then water (30 cm^3^) and CH_2_Cl_2_ (30 cm^3^) were added. The organic layer was separated, and the aqueous layer was washed with diethyl ether (3 × 30 cm^3^). The combined organic layers were washed with water (5 × 50 cm^3^). The resulting solution was dried over magnesium sulfate and concentrated *in vacuo* to afford a red solid. The solid was dissolved in diethyl ether (30 cm^3^) which was washed with 2 M sodium hydroxide (2 × 20 cm^3^), dried over magnesium sulfate, and concentrated *in vacuo* to afford a red solid. The product was further purified through column chromatography to afford **13** (0.46 g, 19%) as a yellow solid. mp 99–101 °C; ^1^H (300 MHz) δ_H_ 7.28 (1H, m), 7.22 (1H, t, *J* 8.4), 7.06 (1H, m), 6.96 (1H, m), 6.62 (1H, d, *J* 8.4), 6.56 (1H, d, *J* 8.7), 5.72 (br t, *J* 4.8, NH), 5.22 (2H, s, OCH_2_), 3.79 (3H, s, OCH_3_), 3.41 (2H, m, NCH_2_), 1.50 (2H, m, CH_2_C*H*_2_CH_2_), 1.34 (2H, m, CH_2_C*H*_2_CH_3_) and 0.89 (3H, t, *J* 7.3, CH_2_C*H*_3_); ^13^C (125 MHz) δ_C_ 165.2 (C=O), 157.4 (C–O), 155.6 (C–O), 138.9 (C), 130.2 (CH), 126.6 (CH), 126.5 (CH), 125.9(CH), 117.1 (C), 105.8 (CH), 104.5 (CH), 65.7 (OCH_2_), 55.8 (OCH_3_), 39.3 (NHCH_2_), 31.4 (CH_2_*C*H_2_CH_2_), 19.8 (CH_2_*C*H_2_CH_3_) and 13.6 (CH_2_*C*H_3_); IR (ATR) ν_max_/cm^−1^ 3387 (NH), 1635. (C=O), 1589, 1541, 1437, 1238, 1086, 760 and 640; HRMS (ESI^+^) calcd. for C_17_H_21_NaNO_3_S (M+Na) 342.1062, found 342.1127.

#### 3.3.4. *N*-Butyl-2-methoxy-6-(2-thienyl(hydroxy)methyl)benzamide **14** and Cyclisation to 7-Methoxy-3-(2-thienyl)isobenzofuran-1(3*H*)-one **15**

A solution of *N*-butyl-2-methoxy-6-(2-thienylmethoxy)benzamide **13** (0.3 g, 1.04 mmol) in dry THF (10 cm^3^) was stirred at RT under nitrogen while *n*-butyllithium (1.8 M in hexanes, 1.92 cm^3^, 3.45 mmol) was added dropwise. After the addition, the mixture was stirred at RT for 15 min then was quenched with saturated aqueous ammonium chloride (10 cm^3^) and the product was extracted with diethyl ether (3 × 20 cm^3^). The organic layer was dried over magnesium sulfate and concentrated *in vacuo* to afford a brown solid (0.16 g, 58%). A portion of the product (16 mg) was further purified through preparative TLC, which resulted in cyclisation to afford **15** (9.7 mg, 30%); ^1^H NMR (300 MHz) δ_H_ 7.63 (1H, t, *J* 7.9, 5-H), 7.35 (1H, dd, *J* = 5.0, *J* = 1.1), 7.14 (1H, d, *J* = 3.5), 7.02–6.97 (3H, m), 6.57 (1H, s, 3-H) and 4.03 (3H, s, OCH_3_); ^13^C (125 MHz) δ_H_ 167.7 (C=O), 158.4 (C), 151.4 (C), 139.1 (C), 136.5 (CH), 127.7 (CH), 127.3 (CH), 127.0 (CH), 114.7 (CH), 113.3 (C), 111.3 (CH), 76.8 (CH) and 56.1 (CH_3_); IR (ATR) ν_max_/cm^−1^ 1759 (C=O), 1601, 1487, 1066 and 704; HRMS (ESI^+^) calcd. for C_13_H_11_O_3_S 247.0429 (M+H), found 247.0415.

#### 3.3.5. Chrycolide **5**

To a stirred solution of 7-methoxy-3-(2-thienyl)isobenzofuran-1(3*H*)-one **15** (8.6 mg, 0.035 mmol) in CH_2_Cl_2_ (5 cm^3^) at rt under N_2_ was added a 1.0 M solution of BBr_3_ (60 μL, 0.070 mmol) in CH_2_Cl_2_, and the solution stirred at rt overnight. The reaction was quenched by the addition of ice-cold H_2_O (5 cm^3^) and the layers separated. The aqueous layer was extracted with CH_2_Cl_2_ (3 × 10 cm^3^), and the combined organic layers dried over MgSO_4_ and concentrated to give, after purification via preparative TLC (hexane/Et_2_O 3:2) at R_f_ 0.11, compound **5** (6.5 mg, 80%) as an orange solid; δ_H_ (500 MHz) 7.59 (1 H, t, *J* 7.9, ArH), 7.40 (1 H, dd, *J* 5.1, 1.2, ArH), 7.17 (1 H, d, *J* 3.4, ArH), 7.04 (1 H, dd, *J* 5.1, 3.6, ArH), 7.01 (1 H, d, *J* 8.3, ArH), 6.96 (1 H, dt, *J* 7.5, 0.8, ArH) and 6.68 (1 H, s, CHO); δ_C_ (125 MHz) 171.3 (C=O), 156.4 (C–O), 148.6 (C), 138.1 (C), 137.2 (CH), 128.3 (CH), 127.9 (CH), 127.1 (CH), 116.2 (CH), 114.6 (CH), 111.1 (C) and 79.0 (CH). The ^1^H and ^13^C spectral data were in accordance with those previously reported [8].

### 3.4. Attempted Synthesis of Isopestacin ***6***

#### 3.4.1. 2,6-Dihydroxy-4-methylbenzoic Acid **17**

Following a literature procedure [14], a stirred solution of orcinol (1.32 g, 9.3 mmol) and KHCO_3_ (2.30 g, 23.0 mmol) in glycerol (2.29 g, 24.9 mmol) was heated to 120 °C for 6 h. The solution was cooled to rt and H_2_O (10 cm^3^) was added. The solution was acidified to pH 1 with conc. HCl and the resulting precipitate filtered off and dried to give **17** (1.28 g, 82%) as a brown solid which was used without further purification; mp (decomp.) 150 °C (lit. [22] 152 °C); δ_H_ (400 MHz) 6.35 (2 H, q, *J* 0.7, ArH) and 2.28 (3 H, t, *J* 0.7, CH_3_). The ^1^H spectral data were in accordance with those previously reported [23].

#### 3.4.2. Methyl 2,6-Dimethoxy-4-methylbenzoate **18**

To a stirred solution of 2,6-dihydroxy-4-methylbenzoic acid **17** (1.00 g, 5.9 mmol) and K_2_CO_3_ (2.40 g, 17.8 mmol) in Me_2_CO (50 cm^3^) was added MeI (2.0 cm^3^, 4.52 g, 31.9 mmol) and the solution heated to reflux for 3 d. The reaction mixture was cooled to rt and filtered, and the filtrate concentrated. The crude residue was dissolved in EtOAc (30 cm^3^) and washed with H_2_O (30 cm^3^) and 2 M NaOH (30 cm^3^). The organic layer was dried over MgSO_4_ and concentrated to give **18** (1.12 g, 90%) as a brown solid, which was used without further purification; mp 74−76 °C (lit. [24] 79−81 °C); δ_H_ (400 MHz) 6.37 (2H, q, *J* 0.7, ArH), 3.88 (3H, s, COOCH_3_), 3.79 (6H, s, 2 × OCH_3_) and 2.34 (3H, t, *J* 0.7, CH_3_); δ_C_ (100 MHz) 167.2 (C=O), 157.1 (2C), 141.7 (C), 110.1 (C), 104.6 (2CH), 55.8 (2CH_3_), 52.2 (CH_3_) and 22.2 (CH_3_). The ^1^H NMR spectral data were in accordance with those previously reported [23]. ^13^C NMR data are reported for the first time.

#### 3.4.3. Methyl 2-Hydroxy-6-methoxy-4-methylbenzoate **19**

To a stirred solution of methyl 2,6-dimethoxy-4-methylbenzoate **18** (1.00 g, 4.76 mmol) in CH_2_Cl_2_ (30 cm^3^) at 0 °C under N_2_ was added BCl_3_ (1.0 M hexanes, 7 cm^3^, 7.00 mmol) and the solution stirred at rt for 1 d. The reaction mixture was poured into ice-water (30 cm^3^) and extracted with CH_2_Cl_2_ (3 × 30 cm^3^). The organic layers were dried over MgSO_4_ and concentrated to give **19** (0.91 g, 97%) as a brown solid, which was used without further purification; mp 86−88 °C (lit. [14] 94−95 °C); δ_H_ (400 MHz) 11.57 (1H, s, OH), 6.43 (1H, m, ArH), 6.23 (1H, m, ArH), 3.94 (3H, s, COOCH_3_), 3.85 (3H, s, OCH_3_) and 2.30 (3H, t, *J* 0.6, CH_3_); δ_C_ (100 MHz) 171.6 (C=O), 163.5 (C–O), 160.6 (C–O), 146.6 (C), 110.5 (CH), 103.5 (CH), 100.4 (C), 56.1 (CH_3_), 52.3 (CH_3_) and 22.2 (CH_3_). The ^1^H and ^13^C NMR spectral data were in accordance with those previously reported [15].

#### 3.4.4. *N*-Butyl-2-hydroxy-6-methoxy-4-methylbenzamide **20**

To a stirred solution of methyl 2-hydroxy-6-methoxy-4-methylbenzoate **19** (0.88 g, 4.5 mmol) in MeOH (15 cm^3^) was added *n*-butylamine (2.31 cm^3^, 1.71 g, 23.4 mmol) and the solution heated under reflux for 8 h. The solution was cooled to rt and concentrated, then acidified to pH1 by the addition of 2 M HCl solution. The aqueous layer was extracted with CH_2_Cl_2_ (3 × 20 cm^3^) and the combined organic layers dried over MgSO_4_ and concentrated to give **20** (0.98 g, 92%) as a light brown solid which was used without further purification; mp 61−63 °C; δ_H_ (400 MHz) 8.26 (1H, s br, NH), 6.44 (1H, dq, *J* 1.5, 0.7, ArH), 6.20 (1H, d, *J* 1.5, ArH), 3.92 (3H, s, OCH_3_), 3.41 (2H, td, *J* 7.1, 5.6, NHC*H*_2_), 2.28 (3H, d, *J* 0.7, CH_3_), 1.64−1.55 (2H, m, NHCH_2_C*H*_2_), 1.45−1.37 (2H, m, C*H*_2_CH_3_) and 0.96 (3H, t, *J* 7.3, CH_2_C*H*_3_); δ_C_ (100 MHz) 170.0 (C=O), 164.2 (C–O), 158.3 (C–O), 144.2 (C), 112.0 (CH), 102.1 (CH), 101.4 (C), 56.0 (OCH_3_), 38.9 (NHCH_2_), 31.4 (NHCH_2_*C*H_2_), 22.0 (CH_3_), 20.2 (*C*H_2_CH_3_) and 13.7 (CH_2_*C*H_3_); ν_max_/cm^−1^ 3393 (N–H), 2959, 1641 (C=O), 1597, 1545 and 1224; HRMS (ESI^+^): calcd. for C_13_H_20_NO_3_ (M+H) 238.1443, found 238.1429.

#### 3.4.5. *N*-Butyl-2-(2,6-dimethoxybenzyloxy)-6-methoxy-4-methylbenzamide **22**

To a solution of *N*-butyl-2-hydroxy-6-methoxy-4-methylbenzamide **20** (0.38 g, 1.62 mmol) in DMF (8 cm^3^), NaH (58 mg, 1.62 mmol) was added, followed after 30 min by a solution of 2,6-dimethoxybenzyl bromide **21** [16] (0.35 g, 1.51 mmol) in DMF (2 cm^3^). The mixture was stirred at rt for 18 h and then added to water (20 cm^3^) and CH_2_Cl_2_ (20 cm^3^). The organic layer was separated, and the aqueous layer extracted with diethyl ether (3 × 20 cm^3^). The combined organic layers were washed with water (5 × 20 cm^3^), then dried and evaporated. Purification via flash column chromatography (gradient elution 1:1 hexane/EtOAc to 100% EtOAc) gave, at R_f_ 0.10, **22** (0.19 g, 32%) as an orange oil ; δ_H_ (400 MHz) 7.26 (1H, t, *J* 8.4, ArH), 6.62−6.52 (3H, m, ArH), 6.37 (1H, s, ArH), 5.99 (1H, t, *J* 5.9, NH), 5.10 (2H, s, OCH_2_), 3.803 (6H, s, 2 × OCH_3_), 3.799 (3H, s, OCH_3_), 3.25 (2H, td, *J* 7.0, 5.9, NHC*H*_2_), 2.34 (3H, s, CH_3_), 1.28−1.22 (2H, m, NHCH_2_C*H*_2_), 1.20−1.13 (2H, m, C*H*_2_CH_3_) and 0.76 (3H, t, *J* 7.2, CH_2_C*H*_3_); δ_C_ (100 MHz) 165.5 (C=O), 159.2 (2 C–O), 158.1 (C–O), 157.1 (C–O), 140.8 (C), 130.1 (CH), 113.0 (C), 112.6 (C), 106.3 (CH), 105.0 (CH), 103.8 (2CH), 60.0 (OCH_2_), 55.9 (OCH_3_), 55.8 (2 OCH_3_), 39.2 (NHCH_2_), 31.3 (NHCH_2_*C*H_2_), 22.1 (CH_3_), 19.8 (*C*H_2_CH_3_) and 13.7 (CH_2_C*H*_3_); HRMS (ESI^+^): calcd. for C_22_H_30_NO_5_ (M+H) 388.2124, found 388.2104.

### 3.5. Attempted Synthesis of Cryphonectric Acid ***7***

#### 3.5.1. 2,6-Dimethoxy-4-methylbenzyl Alcohol **25**

To a stirred suspension of LiAlH_4_ (0.73 g, 19.02 mmol) in dry THF (20 cm^3^), a solution of methyl 2,6-dimethoxy-4-methylbenzoate **18** (1.99 g, 9.47 mmol) in tetrahydrofuran (2 cm^3^) was added dropwise and left to stir. After 2 days, water (7 cm^3^) and 2 M NaOH (7 cm^3^) were added carefully, affording a white precipitate. This precipitate was filtered off under suction using celite, and the filtrate concentrated *in vacuo*. The concentrated filtrate was extracted with CH_2_Cl_2_ (10 cm^3^) and the organic layer was dried over MgSO_4_. The solvent was removed *in vacuo*, affording **25** (1.49 g, 87%) as a yellow solid, which required no further purification; mp 46–48 °C; δ_H_ (300 MHz) 6.38 (2H, s, ArH), 4.74 (2H, s, CH_2_), 3.83 (6H, s, OCH_3_) and 2.34 (3H, s, ArCH_3_). The ^1^H NMR spectral data were found to be consistent with those reported in the literature [25].

#### 3.5.2. 2,6-Dimethoxy-4-methylbenzyl Bromide **26**

To a solution of 2,6-dimethoxy-4-methylbenzyl alcohol **25** (0.5 g, 2.74 mmol) in diethyl ether (8 cm^3^), PBr_3_ (0.107 cm^3^, 0.276 g, 1.02 mmol) was added at 0 °C and stirred for 30 min. Methanol (1.0 cm^3^) and water (10 cm^3^) were added, and the organic layer was collected. The aqueous layer was extracted with ether (8 cm^3^), and the combined organic layers were dried over MgSO_4_. The filtrate was concentrated *in vacuo*, affording **28** (0.67 g, 100%) as an unstable solid. This was immediately dissolved in DMF (8.5 cm^3^) and was directly used for the synthesis of **31**.

#### 3.5.3. Methyl 2,4,6-Trimethoxybenzoate **28**

Following a literature procedure adapted from Barrett and coworkers [26], to a stirred mixture of 2,4,6-trihydroxybenzoic acid **27** (6 g, 32 mmol) and K_2_CO_3_ (26.5 g, 192 mmol) in acetone (100 cm^3^), dimethyl sulfate (18 cm^3^, 190 mmol) was added. The mixture was stirred for 7 days at rt. The reaction was quenched with methanol (7.5 cm^3^) and concentrated aqueous ammonia (2.5 cm^3^) and stirred for 1 h. The mixture was filtered under suction, then concentrated aqueous ammonia (5 cm^3^) and saturated aqueous ammonium chloride (20 cm^3^) were added to the filtrate and stirred for 1 h. The aqueous filtrate was extracted with ether (3 × 30 cm^3^), then the combined organic layers were dried over MgSO_4_, and then concentrated *in vacuo*, affording **28** (5.19 g, 72%) as colourless crystals which required no further purification; mp 65–67 °C (lit. [27] 67–70 °C); δ_H_ (300 MHz) 6.10 (2H, s, ArH), 3.87 (3H, s, OCH_3_), 3.81 (3H, OCH_3_) and 3.79 (6 H, s, OCH_3_); δ_C_ (75 MHz) 166.9 (C=O), 162.5 (C), 158.6 (2C), 90.6 (2CH), 56.0 (OCH_3_), 55.4 (OCH_3_) and 52.2 (OCH_3_). The ^1^H and ^13^C NMR spectral data were found to be consistent with those reported in the literature [28].

#### 3.5.4. Methyl 2-Hydroxy-4,6-dimethoxybenzoate **29**

Following a literature procedure adapted from Barrett and coworkers [26], to a stirred solution of methyl 2,4,6-trimethoxybenzoate **28** (3.83 g, 17.06 mmol) in dry CH_2_Cl_2_ (75 cm^3^) under N_2_ (g), at −78 °C, BCl_3_ (19 cm^3^, 1 M, 19.00 mmol) was added dropwise, affording a cloudy precipitate. The reaction mixture was left to stir at rt overnight, then dilute HCl (45 cm^3^) was added, and the aqueous layer extracted with CH_2_Cl_2_ (3 × 15 cm^3^). The combined organic layers were washed with saturated aqueous NaHCO_3_ (30 cm^3^) and then dried over MgSO_4_. The solvent was then removed *in vacuo*, affording a mixture of **28** and **29**. The crude product was partitioned between ether (30 cm^3^) and 2 M NaOH (30 cm^3^) and the organic and aqueous layers collected. The organic layer was dried over MgSO_4_ and concentrated *in vacuo* to afford **28** (0.72 g). The aqueous layer was neutralised with 2M HCl, then extracted with ethyl acetate (2 × 30 cm^3^), then the combined organic layers were dried over MgSO_4_ and concentrated *in vacuo* to afford **29** (2.26 g, 62%) as yellow crystals which required no further purification; mp 106–109 °C (lit. [27] 107–109 °C); δ_H_ (300 MHz) 12.01 (1H, s, OH), 6.10 (1H, d, *J* 2.4, ArH), 5.95 (1H, d, *J* 2.4, ArH), 3.90 (3H, s, OCH_3_), 3.81 (3H, s, OCH_3_) and 3.79 (3 H, s, OCH_3_); δ_C_ (75 MHz) 171.6 (C=O), 165.9 (C), 165.3 (C), 162.1 (C), 96.5 (C), 93.4 (CH), 91.5 (CH), 56.0 (OCH_3_), 55.4 (OCH_3_) and 52.1 (OCH_3_). The ^1^H NMR spectra data were found to be consistent with those reported in the literature [29].

#### 3.5.5. *N*-Butyl-2-hydroxy-4,6-dimethoxybenzamide **30**

To a stirred solution of methyl 2-hydroxy-4,6-dimethoxybenzoate **29** (2.00 g, 9.4 mmol) in methanol (40 cm^3^), *n*-butylamine (4.55 cm^3^, 3.4 g, 46.0 mmol) was added dropwise, and the solution heated under reflux for 24 h. The solution was cooled to rt, concentrated *in vacuo*, and then acidified to pH 1 by adding 2 M HCl affording crystals. The mixture was extracted with CH_2_Cl_2_ (3 × 40 cm^3^), then the combined organic layers dried over MgSO_4_, and then concentrated *in vacuo* to afford **30** (2.25 g, 94%) as a brown solid requiring no further purification; mp 65–69 °C; δ_H_ (300 MHz) 8.12 (1H, br s, OH), 6.12 (1H, d, *J* 2.4, ArH), 5.95 (1 H, d, *J* 2.4, ArH), 3.89 (3H, s, OCH_3_), 3.78 (3H, s, OCH_3_), 3.40 (2H, td, *J* 7.1, 5.5, NHC*H*_2_), 1.64–1.54 (2H, m, NHCH_2_C*H*_2_), 1.47–1.34 (2H, m, NHCH_2_CH_2_C*H*_2_) and 0.96 (3H, t, *J* 7.3, CH_2_C*H*_3_); δ_C_ (75 MHz) 170.0 (C=O), 166.2 (C), 163.4 (C), 159.7 (C), 97.5 (C), 94.5 (CH), 90.4 (CH), 56.0 (OCH_3_), 55.3 (OCH_3_), 38.8 (NHCH_2_), 31.4 (CH_2_), 20.2 (CH_2_) and 13.7 (CH_3_); ν_max_/cm^−1^ 3391, 2932, 2860, 1640, 1595, 1557, 1393, 1211, 1105, 914 and 820; HRMS (ESI^+^) calcd. for C_13_H_19_NaNO_4_ (M+Na) 276.1212, found 276.1198; calcd. for C_13_H_20_NO_4_ (M+H) 254.1392, found 254.1383.

#### 3.5.6. *N*-Butyl-2,4-dimethoxy-6-(2,6-dimethoxy-4-methylbenzyloxy)benzamide **31**

To a stirred solution of *N*-butyl-2-hydroxy-4,6-dimethoxybenzamide **30** (0.60 g, 2.74 mmol) in DMF (10.0 cm^3^), NaH (104 mg, 2.90 mmol) was added. Then 2,6-dimethoxy-4-methylbenzyl bromide **26** (0.6 g, 2.74 mmol) in DMF (8.5 cm^3^) was added to the reaction mixture and stirred at rt overnight. The reaction mixture was poured into water (30 cm^3^), and CH_2_Cl_2_ (30 cm^3^) was added, then the organic layer was collected. The aqueous layer was then extracted with ether (3 × 30 cm^3^), and the combined organic layers washed with water (5 × 30 cm^3^). The combined organic layers were dried over MgSO_4_ and concentrated *in vacuo*. The product was purified by flash column chromatography and preparative TLC (3:1, hexane: ethyl acetate) to afford, in addition to the starting compound **30** (0.39 g), the impure product **31** (27.5 mg, 2%) as a yellow solid; HRMS (ESI^+^) calcd. for C_23_H_31_NaNO_6_ (M+Na) 440.2049, found 440.2033.

### 3.6. Synthesis 3-Aryl-3-hydroxyisoindolin-1-ones and Their Benzyl Derivatives

#### 3.6.1. Preparation of 2-Butyl-3-hydroxy-3-phenylisoindolin-1(3*H*)-one **34**

A solution of **32** (1.41 g, 4.98 mmol) in dry THF (50 cm^3^) was stirred under nitrogen, while *n*-butyllithium in hexanes (2.5 M, 6.60 cm^3^, 16.4 mmol) was added dropwise. After 2 h, the mixture was added to saturated aq. ammonium chloride (50 cm^3^) and extracted with diethyl ether (3 × 30 cm^3^). Drying and evaporation gave the crude secondary alcohol **33** as an oil. This was taken up in CH_2_Cl_2_ (100 cm^3^) and stirred at rt with manganese dioxide (8.79 g, 0.101 mol) for 24h. The mixture was filtered, and the filtrate evaporated. The crude residue was purified by column chromatography (SiO_2_, Et_2_O/hexane 1:1) to give, at R_f_ 0.45, after subsequent recrystallisation (EtOAc/hexane 1:1), **34** (0.47 g, 34%) as colourless crystals; mp 158–161 °C; (lit. [20] 158–159 °C); δ_H_ (400 MHz) 7.75–7.71 (1H, m, ArH), 7.49–7.26 (8H, m, ArH), 3.66 (1H, s, OH), 3.45–3.35 (1H, m, NC*H*H), 2.99–2.87 (1H, m, NCH*H*), 1.50–1.40 (1H, m, NCH_2_C*H*H), 1.35–1.25 (1H, m, NCH_2_CH*H*), 1.25–1.15 (2H, m, C*H*_2_CH_3_) and 0.78 (3H, t, *J* 7.4, CH_3_); δ_C_ (75 MHz) 167.9 (C=O), 149.1 (C), 138.9 (C), 132.3 (CH), 130.4 (C), 129.1 (CH), 128.3 (3CH), 126.1 (2CH), 123.0 (CH), 122.6 (CH), 91.2 (C(Ph)OH), 39.2 (NCH_2_), 30.5 (NCH_2_*C*H_2_), 20.3 (*C*H_2_CH_3_) and 13.5 (CH_3_); HRMS (ESI^+^): calcd. for C_18_H_19_NaNO_2_ (M+Na) 304.1308, found 304.1302.

#### 3.6.2. 2-Hydroxy-*N*-propylbenzamide **38**

A mixture of methyl salicylate **37** (15.00 g, 98.6 mmol) and *n*-propylamine (40.5 cm^3^, 493.0 mmol) in methanol was heated to reflux for 18 h and concentrated *in vacuo*. The residue was acidified to pH 1 using 2 M HCl(aq) then extracted with CH_2_Cl_2_. The organic layers were washed with water, dried with MgSO_4_ and evaporated to give **38** (16.2 g, 91%) as a waxy peach-coloured oil which was used without further purification. The spectral data were found to be in accordance with previously published data [30]; δ_H_ (400 MHz) 12.47 (1H, s, OH), 7.51 (1H, dd, *J* 8.0, 1.8, ArH), 7.35 (1H, ddd, *J* 8.6, 7.3, 1.6, ArH), 7.06 (1H, s, NH), 6.95 (1H, dd, *J* 8.3, 1.2, ArH), 6.80 (1H, ddd, *J* 8.1, 7.3, 1.2, ArH), 3.43–3.30 (2H, m, NCH_2_), 1.71–1.53 (2H, m, CH_2_) and 0.93 (3H, t, *J* 5.6, CH_3_); δ_C_ (100 MHz) 169.9 (C=O), 160.8 (C–OH), 133.8 (CH), 125.8 (CH), 118.7 (CH), 118.0 (CH), 114.4 (C), 41.2 (NCH_2_), 22.5 (CH_2_) and 11.1 (CH_3_).

#### 3.6.3. 2-Benzyloxy-*N*-propylbenzamide **39**

A solution of benzyl bromide (1.26 cm^3^, 10.6 mmol), 2-hydroxy-*N*-propylbenzamide **38** (1.79 g, 10 mmol) and K_2_CO_3_ (4.19 g, 30.3 mmol) in DMF (10 cm^3^) was heated at 100 °C for 18 h. It was then added to water (50 cm^3^) and extracted with CH_2_Cl_2_ (20 cm^3^) followed by diethyl ether (3 × 20 cm^3^). The combined extract was washed with water (6 × 20 cm^3^), dried, and evaporated. The residue was purified by column chromatography (SiO_2_, EtOAc/hexane 1:1) to yield **39** (1.42 g, 53%) as a colourless oily solid, mp 15–16 °C. δ_H_ (500 MHz) 8.26 (1H, dd, *J* 7.8, 1.9, ArH), 7.93 (1H, s, NH), 7.49–7.36 (6H, m, ArH), 7.14–7.03 (2H, m, ArH), 5.16 (2H, s, OCH_2_), 3.31 (2H, td, *J* 6.9, 5.4, NCH_2_), 1.49–1.32 (2H, m, CH_2_) and 0.75 (3H, t, *J* 7.4, CH_3_); δ_C_ (100 MHz) 164.9 (C=O), 156.6 (C–O), 135.3 (C), 132.3 (CH), 132.0 (CH), 128.6 (2CH), 128.5 (CH), 127.9 (2CH), 121.5 (C), 121.2 (CH), 112.2 (CH), 71.1 (OCH_2_), 41.2 (NCH_2_), 22.1 (CH_2_) and 11.1 (CH_3_). HRMS (ESI^+^): calcd. for C_17_H_19_NO_2_ (M+H) 270.1494, found 270.1482.

#### 3.6.4. 2-(4-Chlorobenzyloxy)-*N*-propylbenzamide **40**

The method of 3.6.3 was followed using 4-chlorobenzyl chloride (1.71 g, 10.6 mmol), 2-hydroxy-*N*-propylbenzamide **38** (1.79 g, 10 mmol) and K_2_CO_3_ (4.19 g, 30.3 mmol) in DMF (10 cm^3^). After extraction, washing, drying and evaporation, the product was obtained without further purification to yield **40** (2.83 g, 93%) as a colourless solid, mp 130–132 °C; δ_H_ (400 MHz) 8.24 (1 H, dd, *J* = 7.8, 1.9 Hz, ArH), 7.78 (1 H, s, NH), 7.45–7.34 (5 H, m, ArH), 7.11 (1 H, t, *J* = 6.8 Hz, ArH), 7.02 (1 H, d, *J* = 7.7 Hz, ArH), 5.14 (2 H, s, OCH_2_), 3.34 (2 H, td, *J* = 6.9, 5.5 Hz, NCH_2_), 1.48–1.36 (2 H, m, CH_2_) and 0.78 (3 H, t, *J* = 7.4 Hz, CH_3_); δ_C_ (100 MHz) 165.3 (C=O), 156.8 (C-O), 138.4 (C), 134.4 (C-Cl), 132.9 (CH), 132.8 (CH), 129.8 (2CH), 129.5 (2CH), 122.4 (C), 122.2 (CH), 70.9 (OCH_2_), 41.8 (NCH_2_), 22.8 (CH_2_) and 11.7 (CH_3_). HRMS (ESI^+^): calcd. for C_17_H_19_^35^ClNO_2_ (M+H) 304.1104, found 304.1096.

#### 3.6.5. 2-(Hydroxy(phenyl)methyl)-*N*-propylbenzamide **41**

A solution of **39** (0.539 g, 2.00 mmol) in dry THF (20 cm^3^) was stirred under nitrogen while *n*-butyllithium in hexanes (2.5 M, 2.60 cm^3^, 6.50 mmol) was added dropwise. After 2 h the mixture was added to saturated aq. ammonium chloride (20 cm^3^) and extracted with diethyl ether (3 × 10 cm^3^). Drying and evaporation gave **41** (0.440 g, 82%) as a brown viscous oil; δ_H_ (400 MHz) 7.47–7.19 (9H, m, ArH), 6.48 (1H, s, NH), 5.87 (1H, s, C***H***OH), 3.20–3.04 (2H, m, NCH_2_), 1.35–1.27 (2H, m, CH_2_) and 0.81 (3H, t, *J* 8.8, CH_3_); δ_C_ (100 MHz) 170.8 (C=O), 143.0 (C), 142.7 (C), 135.8 (C), 130.5 (CH), 129.8 (CH), 127.74 (CH), 127.69 (2CH), 127.60 (CH), 126.6 (CH), 126.2 (2CH), 74.7 (CH–O), 41.6 (NCH_2_), 22.2 (CH_2_) and 11.2 (CH_3_).

#### 3.6.6. 2-(Hydroxy(4-chlorophenyl)methyl)-*N*-propylbenzamide **42**

The method of 3.6.5 using *n*-butyllithium (2.60 cm^3^, 6.50 mmol), and compound **40** (0.608 g, 2.00 mmol) in dry THF (20 cm^3^) gave **42** (0.610 g, 100%) as a red sticky oil. δ_H_ (400 MHz) 7.42–7.18 (8H, m, ArH), 6.37 (1H, br t, *J* 5.5, NH), 5.93 (1H, s, OH), 5.76 (1H, s, CH–O), 3.26–3.13 (1H, m, NC*H**_A_***H), 3.13–3.01 (1H, m, NCH*H**_B_***), 1.36–1.28 (2H, m, CH_2_) and 0.80 (3H, t, *J* 7.4, CH_3_); δ_C_ (100 MHz) 170.9 (C=O), 143.2 (C), 141.6 (C), 135.7 (C), 132.5 (C), 130.9 (CH), 130.3 (CH), 128.00 (CH), 127.85 (2CH), 127.76 (2CH), 127.69 (CH), 74.8 (CH–O), 41.8 (NCH_2_), 22.4 (CH_2_) and 11.2 (CH_3_).

#### 3.6.7. 3-Hydroxy-3-phenyl-2-propylisoindolin-1(3*H*)-one **43**

A solution of **41** (580 mg, 2.15 mmol) in CH_2_Cl_2_ (40 cm^3^) was stirred at rt with manganese dioxide (3.81 g, 43.8 mmol) for 24h. The mixture was filtered and the filtrate evaporated. The crude residue was purified by preparative TLC (SiO_2_, Et_2_O/hexane 1:1) to yield **43** (340 mg, 59%) as a yellow solid; mp 95–102 °C. δ_H_ (500 MHz, CDCl_3_) 7.71 (1H, d, *J* 7.3, ArH), 7.50–7.23 (8H, m, ArH), 3.39 (1H, ddd, *J* 14, 10, 5.5, NC*H*_A_), 2.91 (1H, ddd, *J* 14, 10, 5.5, NC*H*_B_), 1.52 (1H, dddq, *J* 13, 10, 7.5, 5.5, CH*H**_C_***), 1.39 (1H, dddq, *J* 13, 10, 7.5, 5.5, C*H_D_*H) and 0.78 (3H, t, *J* 7.5, CH_3_). Coupling values derived by simulation to 3 decimal place shift accuracy—see Table 1. δ_C_ (100 MHz, CDCl_3_) 167.7 (C=O), 148.9 (C), 138.6 (C), 132.5 (CH), 130.6 (C), 129.4 (CH), 128.45 (2CH), 128.40 (CH), 126.1 (2CH), 123.2 (CH), 122.6 (CH), 91.4 (C–OH), 41.3 (NCH_2_), 22.1 (CH_2_) and 11.7 (CH_3_). The NMR spectroscopic data agree with literature values [21].

#### 3.6.8. 3-(4-Chlorophenyl)-3-hydroxy-2-propylisoindolin-1(3*H*)-one **44**

The method of 3.6.7 using manganese dioxide (3.53 g, 40.6 mmol) and **42** (608 mg, 2.00 mmol) in CH_2_Cl_2_ (40 cm^3^) followed by column chromatography (SiO_2_, Et_2_O/hexane 1:1) gave **44** (210 mg, 35%) as a peach solid, mp 201–202 °C (lit. [21] 188–190 °C). δ_H_ (400 MHz) 7.78–7.75 (1H, m, ArH), 7.49 (1H, td, *J* 7.6, 1.2, ArH), 7.46 (1H, td, *J* 7.2, 1.2, ArH), 7.37–7.27 (4H, m, ArH), 7.27–7.21 (1H, m, ArH), 3.41 (1H, ddd, *J* 13.9, 10.0, 5.8, NC*H_A_*H), 3.17 (1H, s, OH), 2.92 (1H, ddd, *J* 13.9, 10.0, 5.7, NCH*H_B_*), 1.58–1.35 (2H, m, CH_2_) and 0.81 (3H, t, *J* 7.4, CH_3_); δ_C_ (100 MHz) 167.6 (C=O), 148.4 (C), 137.2 (C), 134.5 (C), 132.7 (CH), 130.5 (C), 129.8 (CH), 128.7 (2CH), 127.7 (2CH), 123.4 (CH), 122.5 (CH), 91.0 (C–OH), 41.3 (NCH_2_), 22.2 (CH_2_) and 11.7 (CH_3_). The NMR spectroscopic data agree with the literature values [21].

#### 3.6.9. 3-(4-Hydroxy-3,5-dimethoxybenzyloxy)-3-phenyl-2-propylisoindolin-1(3*H*)-one **35**

Thionyl chloride (0.278 cm^3^, 1.50 mmol) was added to a solution of **43** (200 mg, 0.75 mmol) in THF (10 cm^3^) with a catalytic amount of DMF, and the mixture was stirred at rt for 16 h before evaporating off the solvent. The residue was taken up in DMF (10 cm^3^) and 3,5-dimethoxy-4-hydroxybenzyl alcohol (276 mg, 1.5 mmol) and triethylamine (0.21 cm^3^, 1.5 mmol) were added. After stirring at rt for 20 h, the mixture was added to water (50 cm^3^) and extracted with ethyl acetate (3 × 20 cm^3^). The combined extracts were washed with water (6 × 30 cm^3^), dried and evaporated. The crude product was purified by preparative TLC (SiO_2_, EtOAc/hexane 1:1) to yield **35** (10 mg, 5%) as a yellow oil. The spectral data were found to be in accordance with previously published data [6]. δ_H_ (400 MHz) 8.17 (1H, s, OH), 7.72–7.70 (1 H, m, ArH), 7.43–7.20 (8H, m, ArH), 6.60 (2H, s, ArH), 4.50 (2H, s, OCH_2_), 3.78 (6H, s, OMe), 3.43–3.35 (1 H, m, NC*H_A_*H), 2.91–2.84 (1H, m, NCH*H_B_*), 1.53–1.35 (2H, m, CH_2_) and 0.75 (3H, t, *J* 7.4, CH_3_); δ_C_ (100 MHz) 167.6 (C=O), 152.0 (2C), 148.8 (C), 146.7 (C), 138.5 (C), 136.3 (C), 132.5 (CH), 130.6 (C), 129.6 (CH), 128.5 (2CH), 128.4 (CH), 126.1 (2CH), 123.3 (CH), 122.5 (CH), 105.1 (2CH), 91.4 (C), 56.2 (2OMe), 46.3 (OCH_2_), 41.4 (NCH_2_), 22.2 (CH_2_) and 11.7 (CH_3_).

#### 3.6.10. 3-(4-Chlorophenyl)-3-(4-hydroxy-3,5-dimethoxybenzyloxy)-2-propylisoindolin-1(3*H*)-one **36**

The method of 3.6.9 using thionyl chloride (0.278 cm^3^, 1.50 mmol) and **44** (200 mg, 0.66 mmol) in THF (10 cm^3^) with a catalytic amount of DMF followed by triethylamine (0.21 cm^3^, 1.5 mmol) and 3,5-dimethoxy-4-hydroxybenzyl alcohol (276 mg, 1.5 mmol) followed by preparative TLC (SiO_2_, EtOAc/hexane 1:1) gave **36** (150 mg, 49%) as an orange oil. The spectral data were found to be in accordance with previously published data [6]. δ_H_ (400 MHz) 7.92–7.88 (1H, m, ArH), 7.52 (1H, td, *J* 8.0, 1.2, ArH), 7.48 (1H, td, *J* 8.0, 1.6, ArH), 7.35 and 7.30 (4H, AB pattern, *J* 8.8, 4ArH), 7.14–7.10 (1H, m, ArH), 6.46 (2H, s, 2ArH), 5.60 (1H, br s, OH), 4.15 (1H, d, *J* 11.4, OC*H_A_*H), 3.92 (1H, d, *J* 11.4, OCH*H_B_*) 3.35–3.26 (1H, m, NC*H_A_*H), 3.16–3.06 (1H, m, NC*H_B_*H), 1.62–1.46 (1H, m, C*H_A_*H), 1.46–1.33 (1H, m, C*H_B_*H) and 0.82 (3H, t, *J* 7.2, CH_3_); δ_C_ (100 MHz) 168.2 (C=O), 146.8 (2C), 145.1 (C), 137.5 (C), 134.34 (C), 134.27 (C), 132.4 (CH), 131.8 (C), 129.8 (CH), 128.5 (2CH), 128.2 (C), 127.8 (2CH), 123.4 (CH), 123.1 (CH), 104.4 (2CH), 94.7 (C), 65.2 (OCH_2_), 56.2 (2OMe), 41.4 (NCH_2_), 21.6 (CH_2_) and 11.7 (CH_3_).

## 4. Conclusions

The application of our [1,2]-Wittig rearrangement approach to target syntheses of selected 3-arylphthalide natural products has revealed some limitations. While the method was successful in the case of crycolide, providing the only second synthesis of this compound, extension to examples such as isopestacin and cryphonectric acid bearing 2,6-disubstituted aryl groups failed, most likely due to the steric hindrance blocking either the alkylation or cyclisation stage. On the other hand, oxidative treatment of the crude Wittig rearrangement products provides a convenient new route to 3-aryl-3-hydroxyisoindolinones, and this allowed the synthesis of two anticancer compounds.

## Figures and Tables

**Table 1:** Chemical shift values (ppm) and coupling constants (Hz) used to achieve the simulation of Figure 3.

Proton (*)	δ	J_*–H1b_	J_*–H2a_	J_*–H2b_	J_*–H3_
H-1a	3.394	14	10	5.5	—
H-1b	2.912		5.5	10	—
H-2a	1.517			13	7.5
H-2b	1.393				7.5
3 × H-3	0.785				

## Data Availability

The research data supporting this publication can be accessed at https://doi.org/10.17630/10d97ec3-6df2-4c00-80f2-7b814fc3c99e.

## Supplementary Materials

The following supporting information can be downloaded at: https://www.mdpi.com/article/10.3390/molecules29194722/s1, Figures S1–S36: ^1^H and ^13^C NMR spectra of all new compounds and target products **5**, **35** and **36**.
