# Supplementary material for: Application of the Wittig Rearrangement of N-Butyl-2-benzyloxybenzamides to Synthesis of Phthalide Natural Products and 3-Aryl-3-benzyloxyisoindolinone Anticancer Agents
Source: Molecules. 2024 Oct 6;29(19):4722. doi: 10.3390/molecules29194722 (PMC11478121; doi:10.3390/molecules29194722)

Application of the Wittig Rearrangement of *N*-Butyl-2-benzyloxybenzamides to Synthesis of Phthalide Natural Products and 3-Aryl-3-benzyloxyisoindolinone Anticancer Agents

R. Alan Aitken *, Francesca K. Cooper, Andrew D. Harper, Ryan A. Inwood, Elizabeth A. Saab and Ewan J. Soutar

Supplementary Material

Contents

Figure S1: 500 MHz ^{1}H NMR spectrum of **9** ($CDCl_3$)

Figure S2: 125 MHz ^{13}C NMR spectrum of **9** ($CDCl_3$)

Figure S3: 300 MHz ^{1}H NMR spectrum of **11** ($CDCl_3$)

Figure S4: 125 MHz ^{13}C NMR spectrum of **11** ($CDCl_3$)

Figure S5: 300 MHz ^{1}H NMR spectrum of **13** ($CDCl_3$)

Figure S6: 125 MHz ^{13}C NMR spectrum of **13** ($CDCl_3$)

Figure S7: 500 MHz ^{1}H NMR spectrum of **15** ($CDCl_3$)

Figure S8: 125 MHz ^{13}C NMR spectrum of **15** ($CDCl_3$)

Figure S9: 500 MHz ^{1}H NMR spectrum of **5** ($CDCl_3$)

Figure S10: 125 MHz ^{13}C NMR spectrum of **5** ($CDCl_3$)

Figure S11: 100 MHz ^{13}C NMR spectrum of **18** ($CDCl_3$)

Figure S12: 100 MHz ^{13}C NMR spectrum of **19** ($CDCl_3$)

Figure S13: 400 MHz ^{1}H NMR spectrum of **20** ($CDCl_3$)

Figure S14: 100 MHz ^{13}C NMR spectrum of **20** ($CDCl_3$)

Figure S15: 400 MHz ^{1}H NMR spectrum of **22** ($CDCl_3$)

Figure S16: 100 MHz ^{13}C NMR spectrum of **22** ($CDCl_3$)
Figure S17: 300 MHz ^{1}H NMR spectrum of **30** ($CDCl_3$)
Figure S18: 75 MHz ^{13}C NMR spectrum of **30** ($CDCl_3$)
Figure S19: 400 MHz ^{1}H NMR spectrum of **34** ($CDCl_3$)
Figure S20: 75 MHz ^{13}C NMR spectrum of **34** ($CDCl_3$)
Figure S21: 500 MHz ^{1}H NMR spectrum of **39** ($CDCl_3$)
Figure S22: 100 MHz ^{13}C NMR spectrum of **39** ($CDCl_3$)
Figure S23: 400 MHz ^{1}H NMR spectrum of **40** ($CDCl_3$)
Figure S24: 100 MHz ^{13}C NMR spectrum of **40** ($CDCl_3$)
Figure S25: 400 MHz ^{1}H NMR spectrum of **41** ($CDCl_3$)
Figure S26: 100 MHz ^{13}C NMR spectrum of **41** ($CDCl_3$)
Figure S27: 400 MHz ^{1}H NMR spectrum of **42** ($CDCl_3$)
Figure S28: 100 MHz ^{13}C NMR spectrum of **42** ($CDCl_3$)
Figure S29: 500 MHz ^{1}H NMR spectrum of **43** ($CDCl_3$)
Figure S30: 100 MHz ^{13}C NMR spectrum of **43** ($CDCl_3$)
Figure S31: 400 MHz ^{1}H NMR spectrum of **44** ($CDCl_3$)
Figure S32: 100 MHz ^{13}C NMR spectrum of **44** ($CDCl_3$)
Figure S33: 400 MHz ^{1}H NMR spectrum of **35** ($CDCl_3$)
Figure S34: 100 MHz ^{13}C NMR spectrum of **35** ($CDCl_3$)
Figure S35: 400 MHz ^{1}H NMR spectrum of **36** ($CDCl_3$)
Figure S36: 100 MHz ^{13}C NMR spectrum of **36** ($CDCl_3$)

Figure S1: 500 MHz ^{1}H NMR spectrum of **9** ($CDCl_3$)

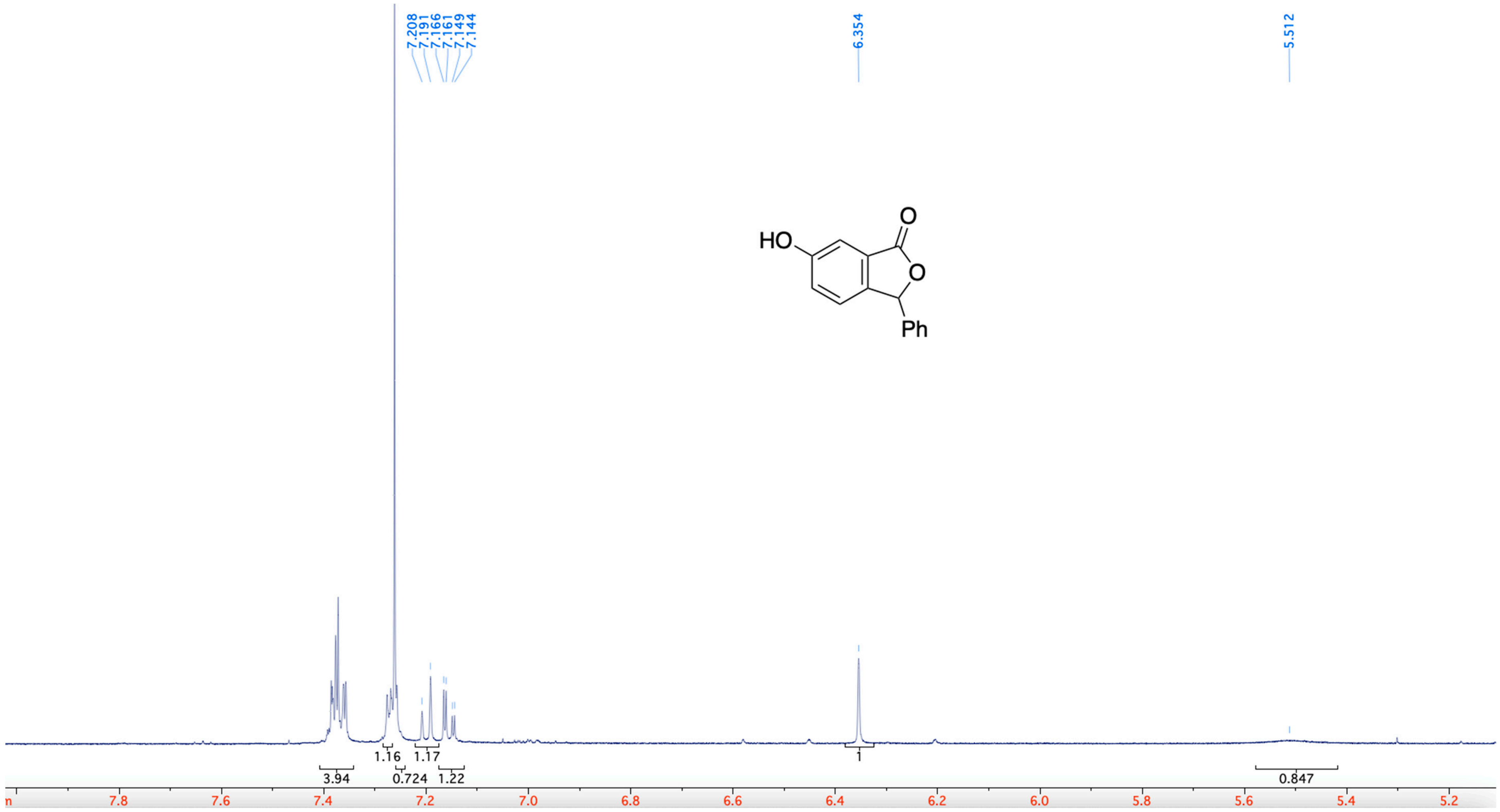

Figure S2: 125 MHz ^{13}C NMR spectrum of **9** ($CDCl_3$)

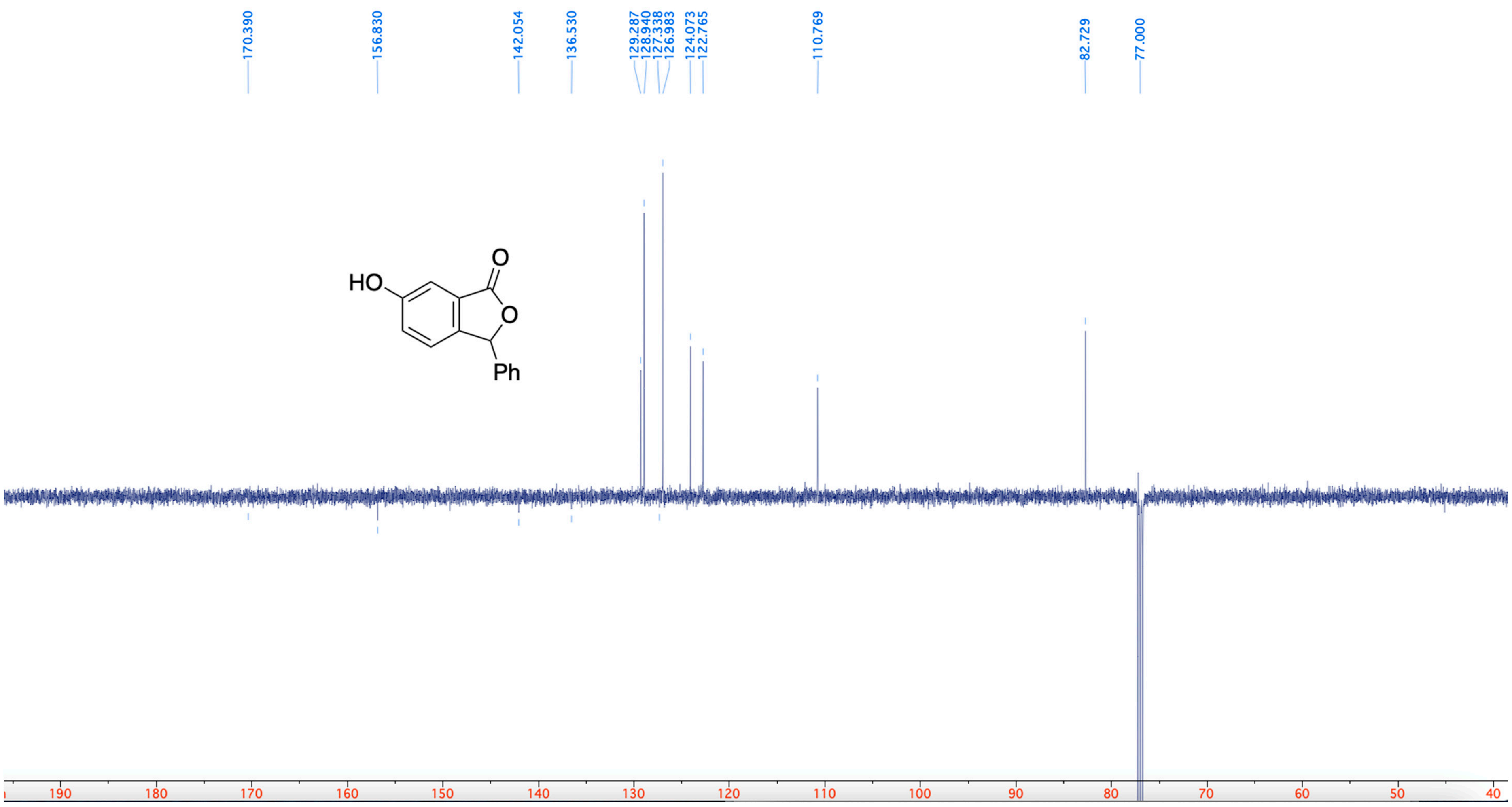

Figure S3: 300 MHz ^{1}H NMR spectrum of **11** ($CDCl_3$)

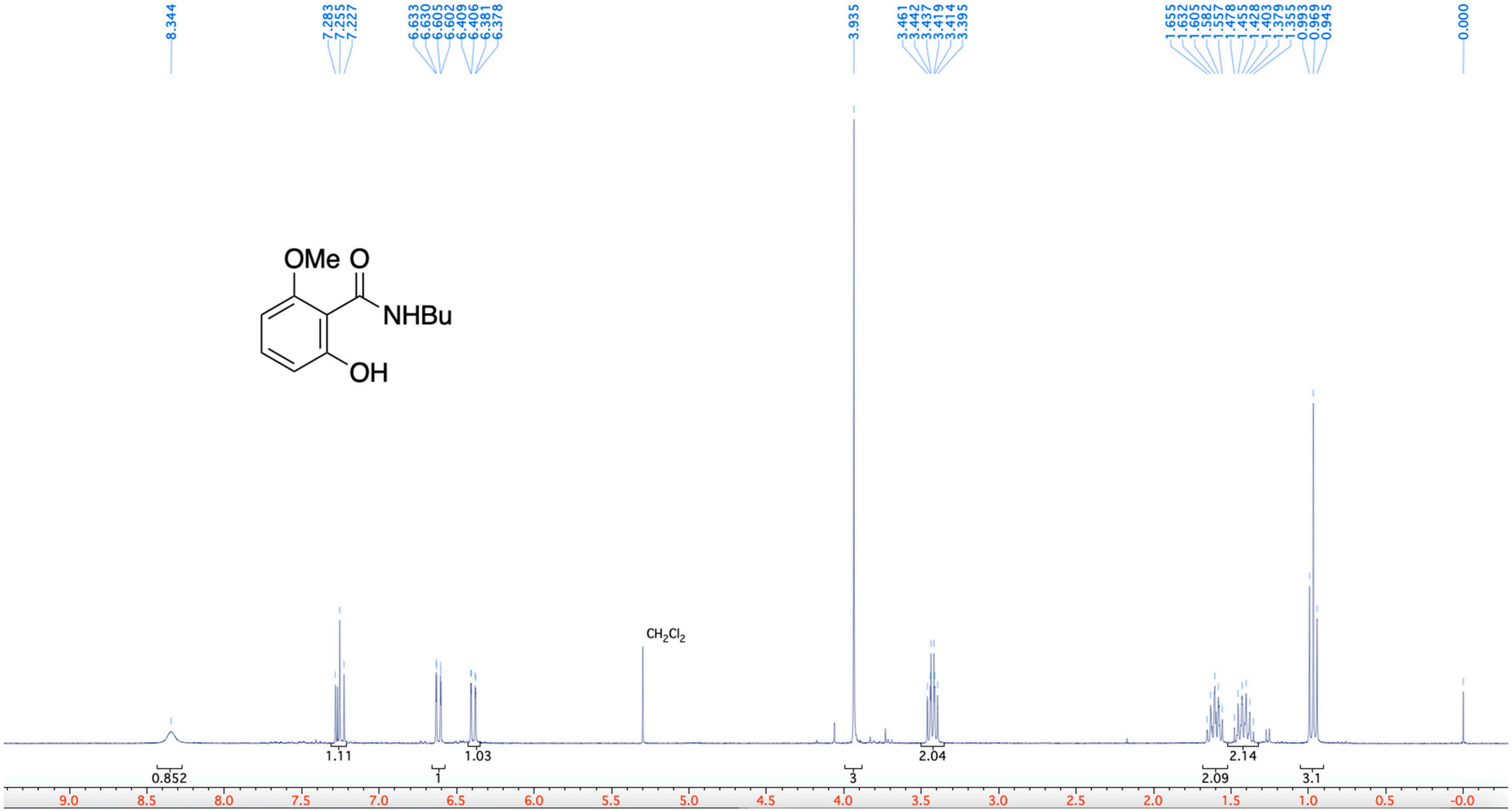

Figure S4: 125 MHz ^{13}C NMR spectrum of **11** ($CDCl_3$)

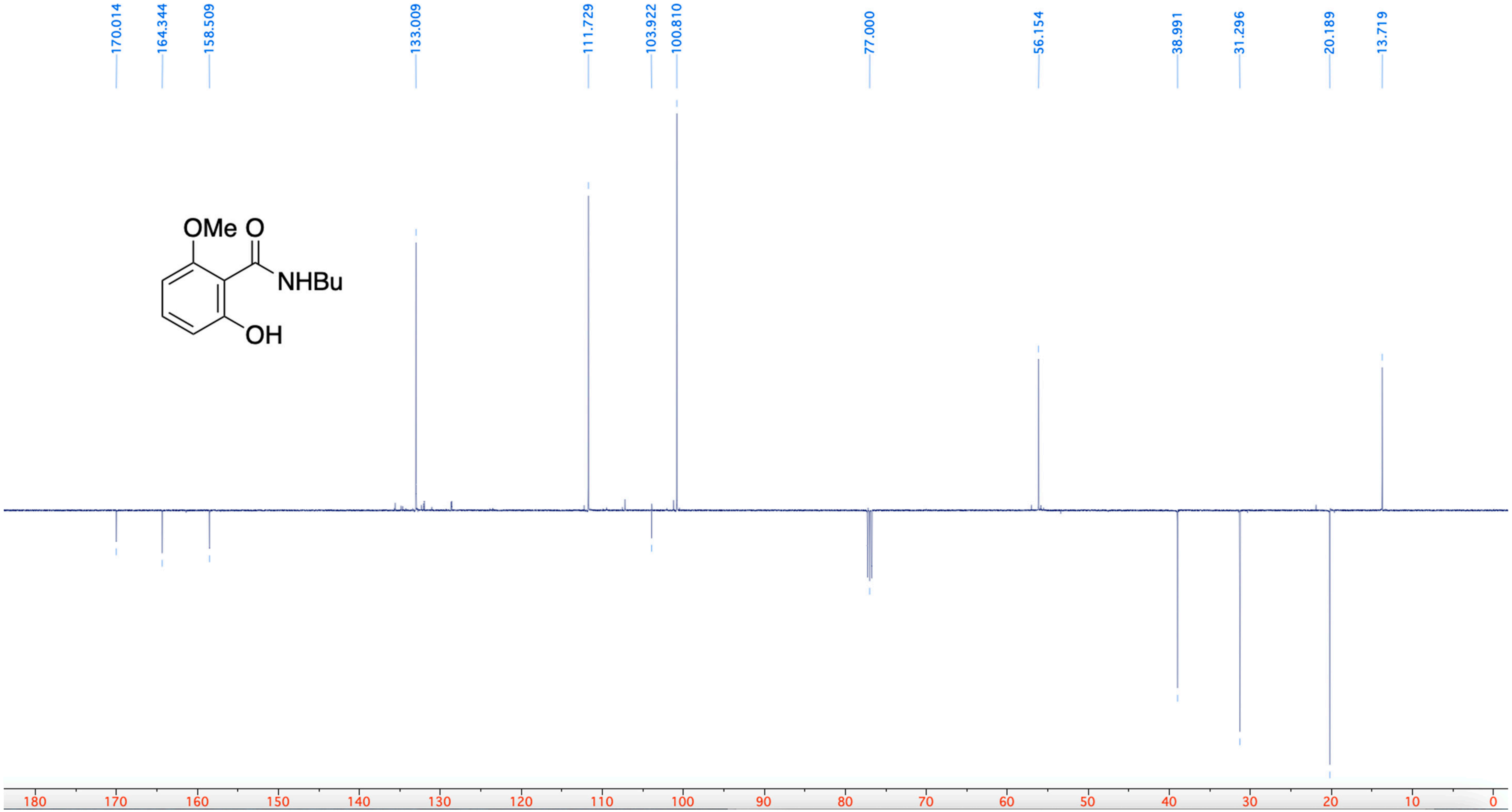

Figure S5: 300 MHz ^{1}H NMR spectrum of **13** ($CDCl_3$)

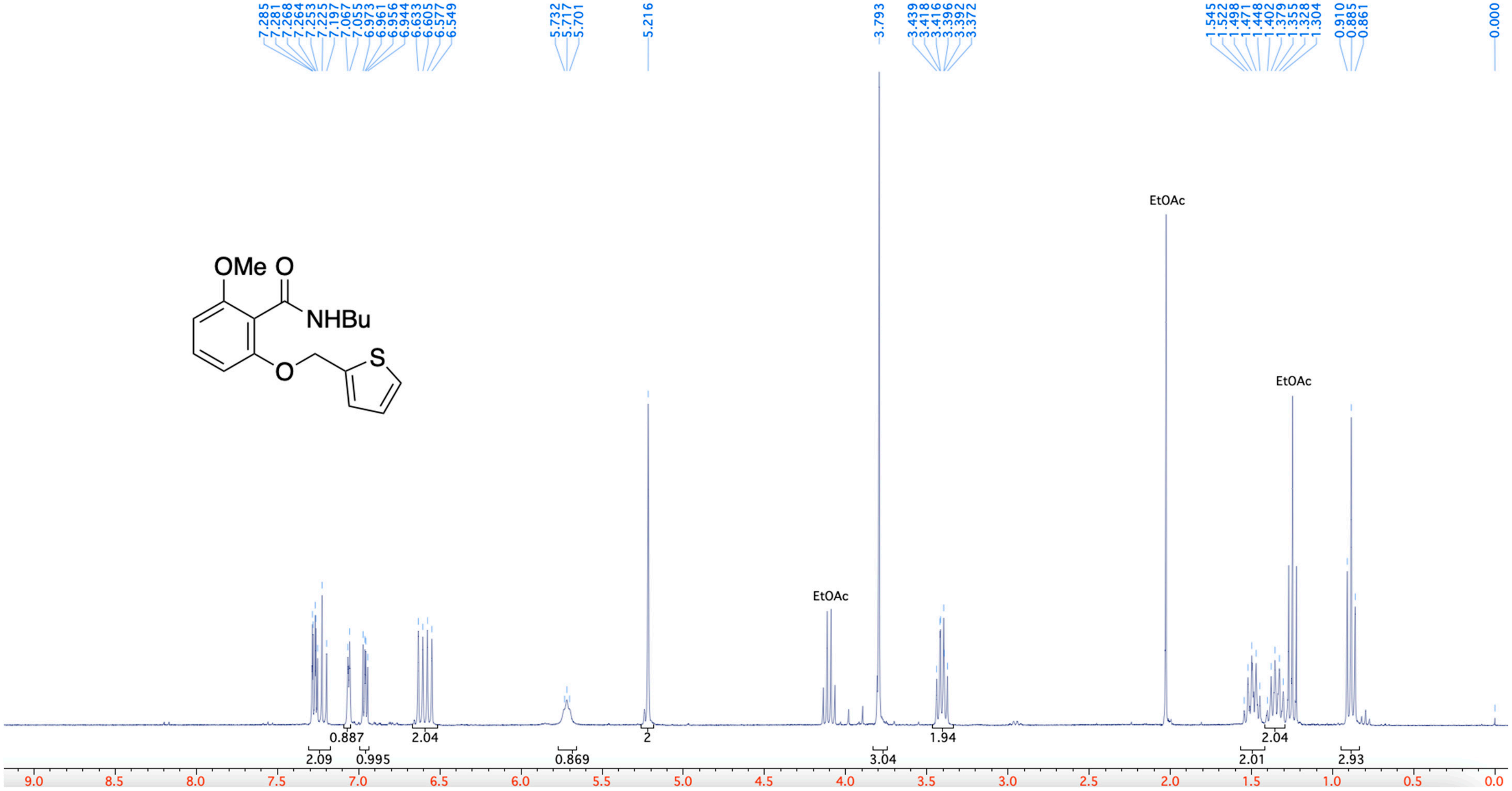

Figure S6: 125 MHz ^{13}C NMR spectrum of **13** ($CDCl_3$)

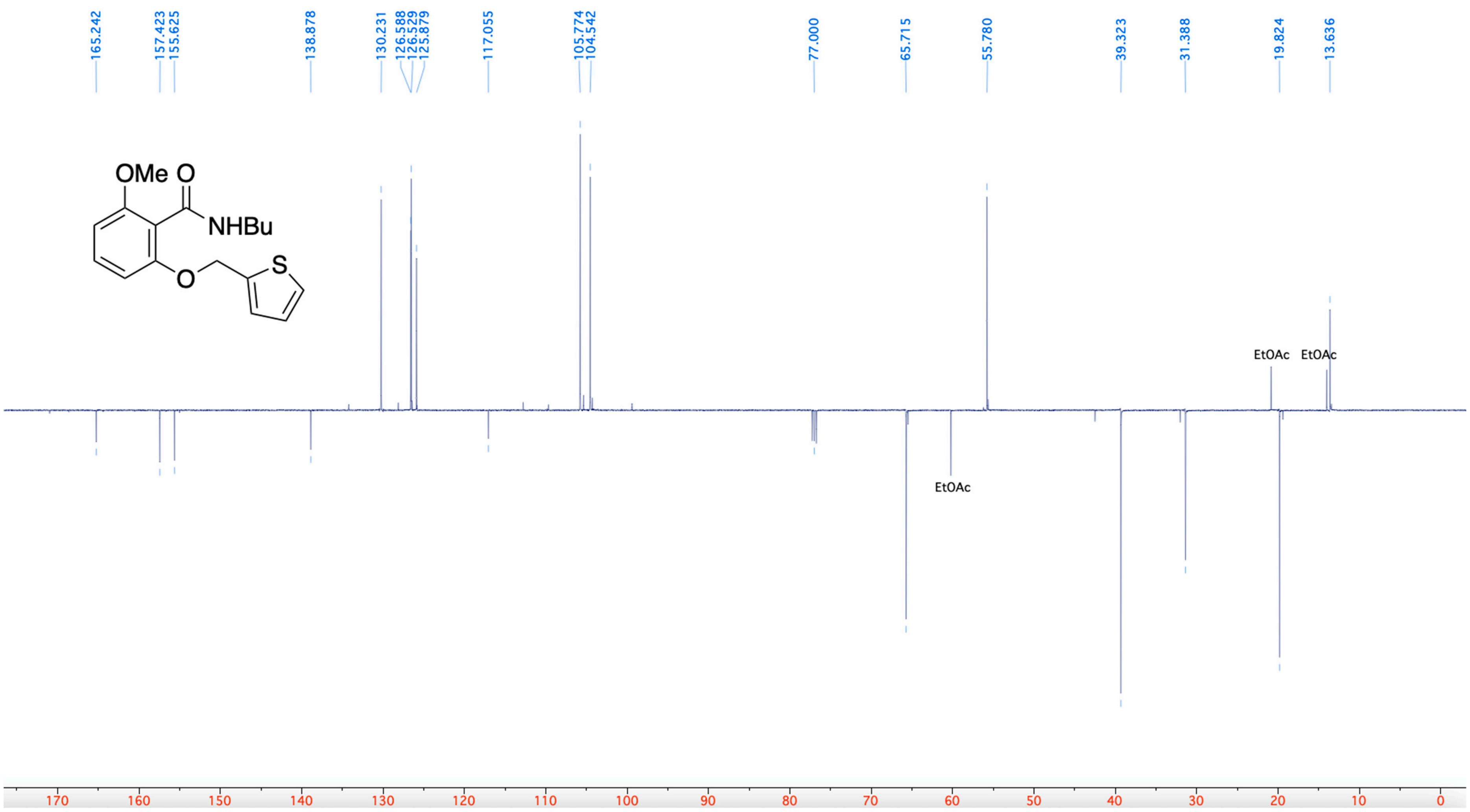

Figure S7: 500 MHz ^{1}H NMR spectrum of **15** ($CDCl_3$)

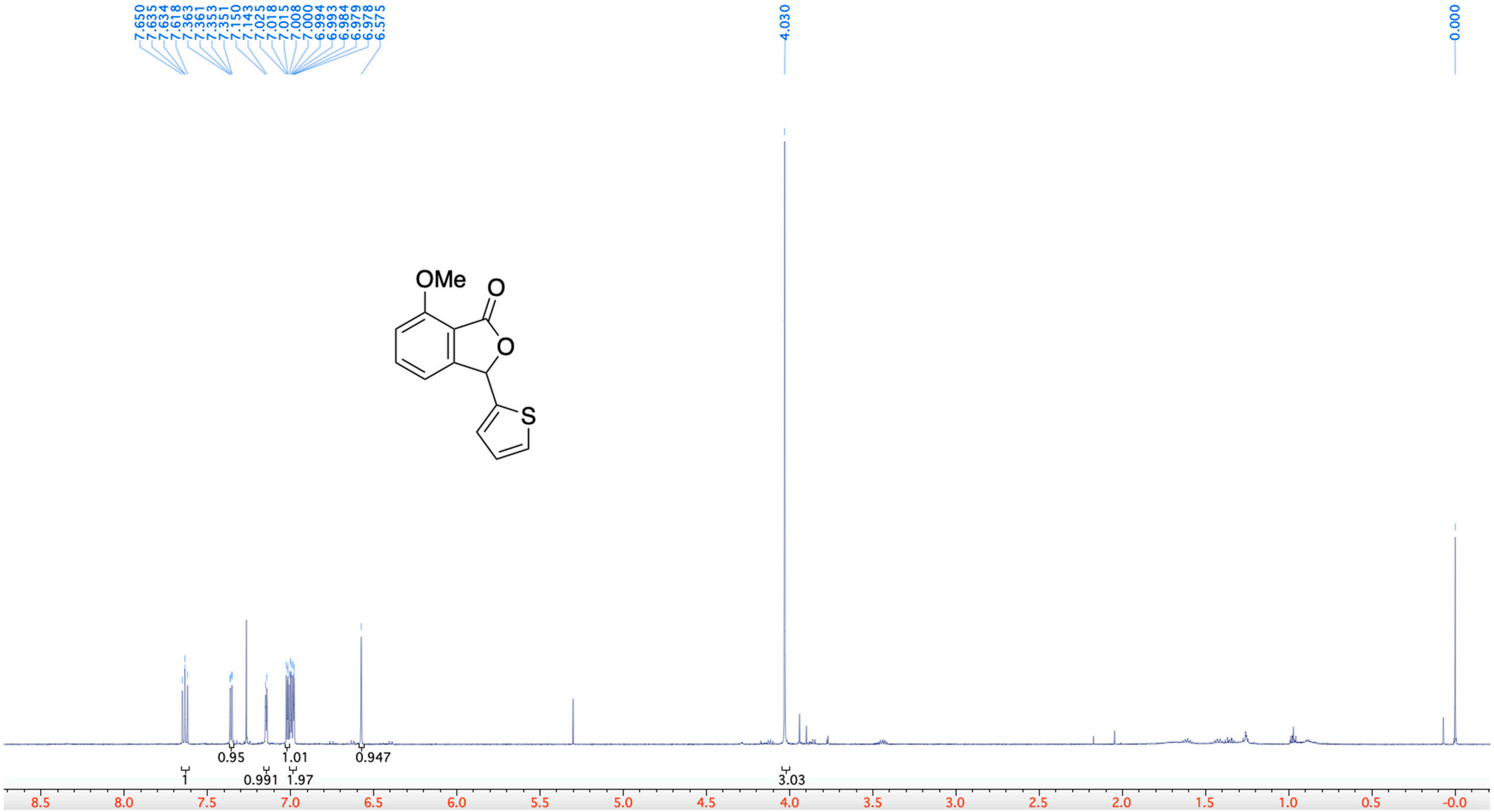

Figure S8: 125 MHz ^{13}C NMR spectrum of **15** ($CDCl_3$)

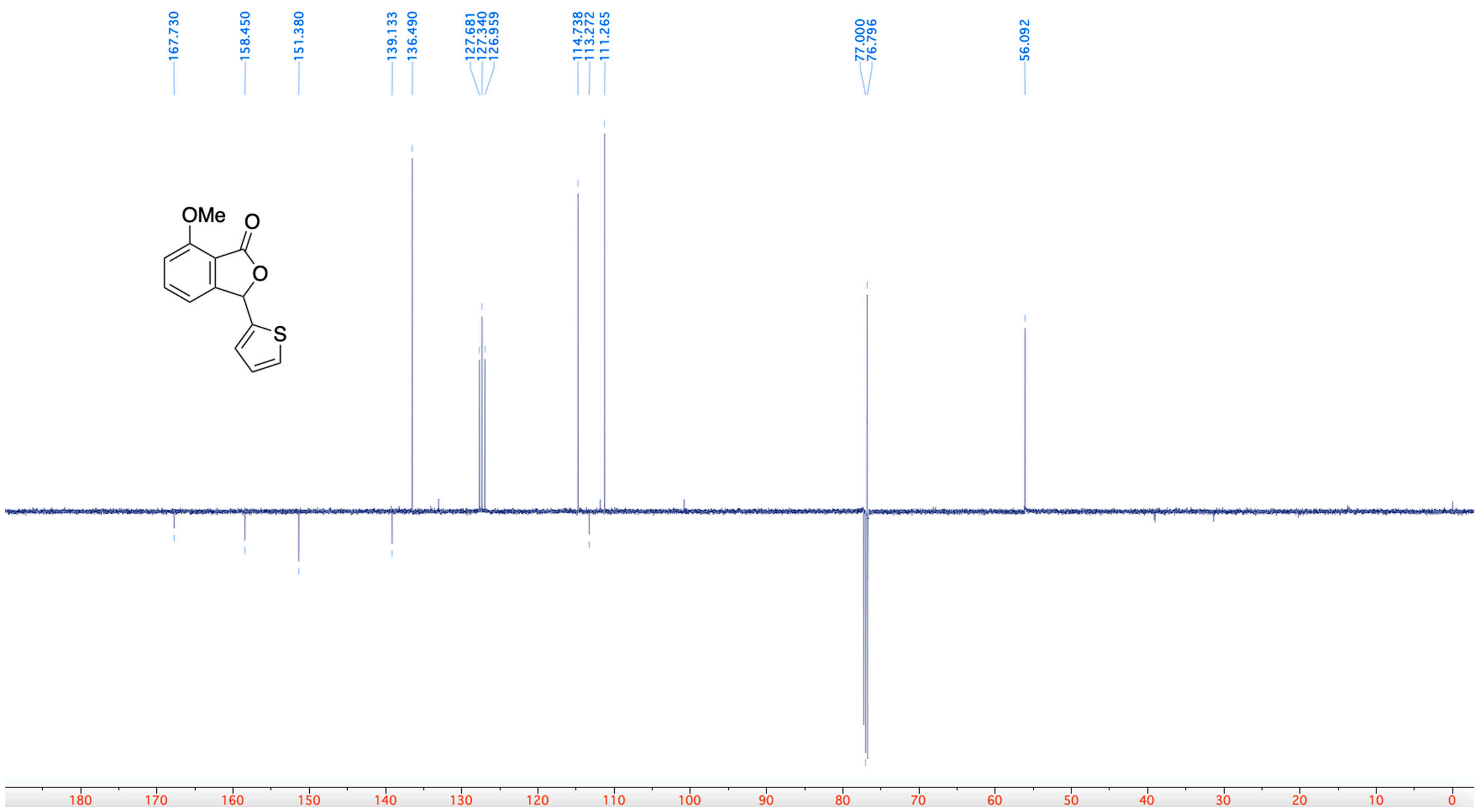

Figure S9: 500 MHz ^{1}H NMR spectrum of **5** ($CDCl_3$)

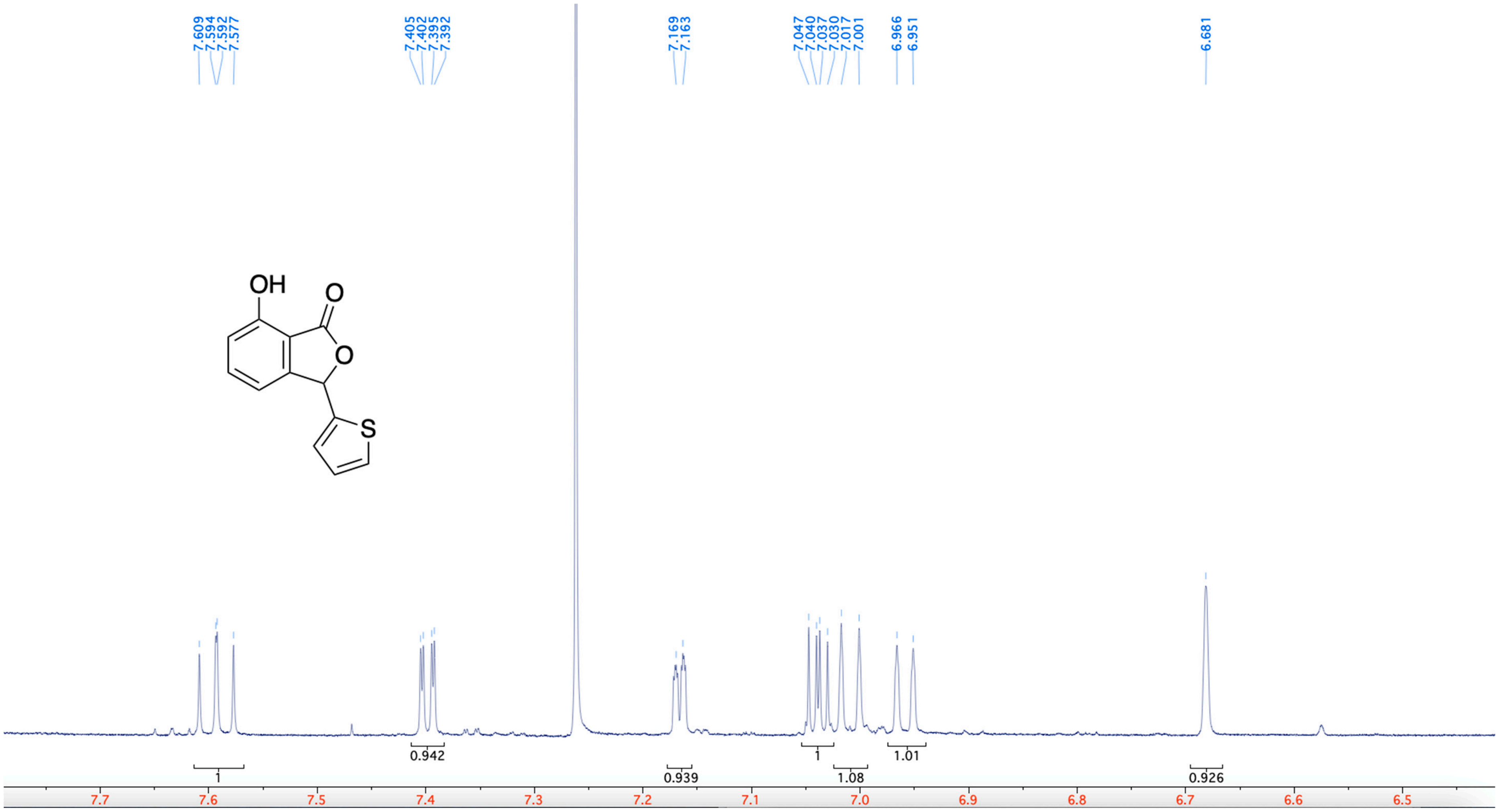

Figure S10: 125 MHz ^{13}C NMR spectrum of **5** ($CDCl_3$)

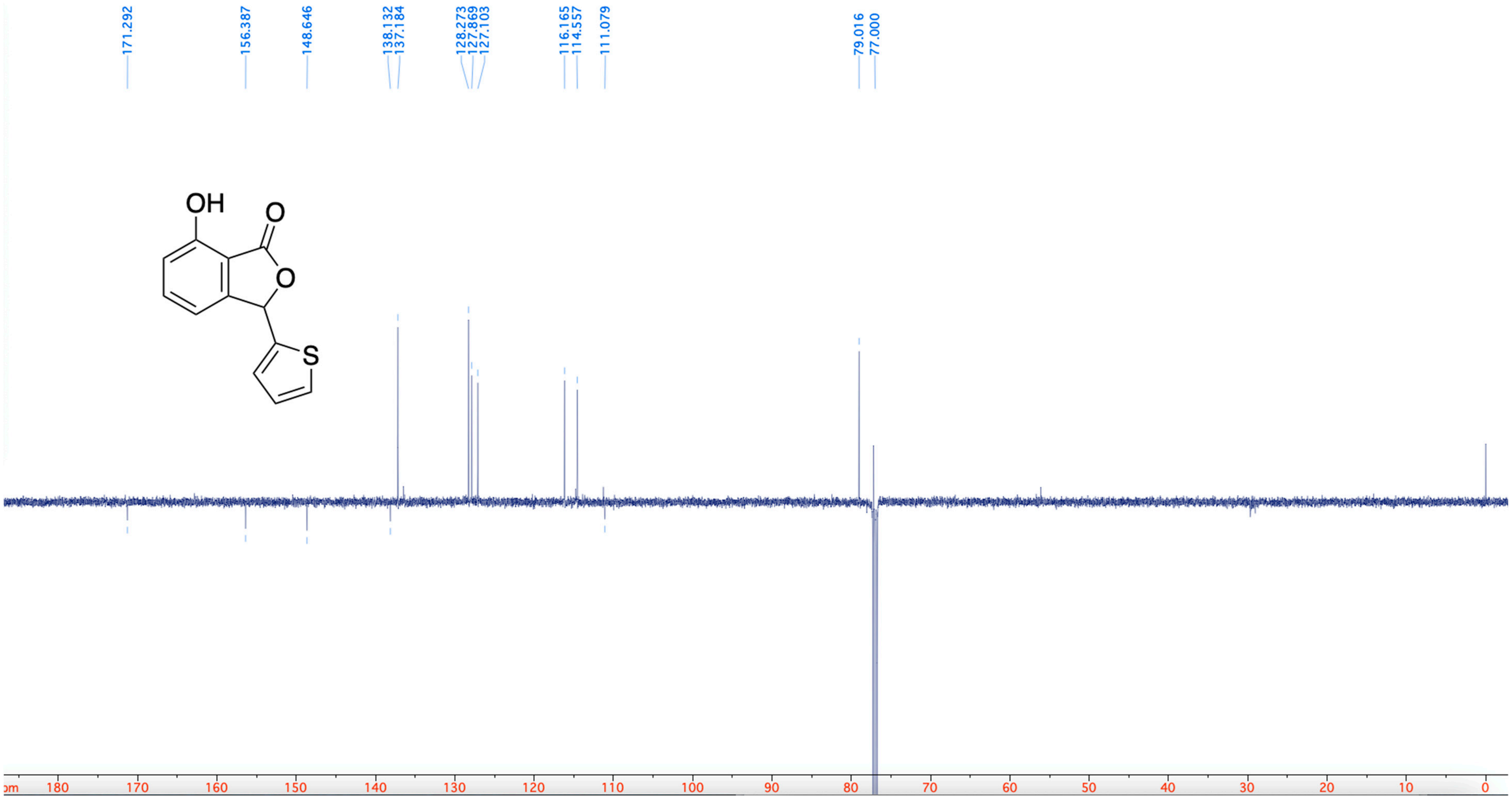

Figure S11: 100 MHz ^{13}C NMR spectrum of **18** ($CDCl_3$)

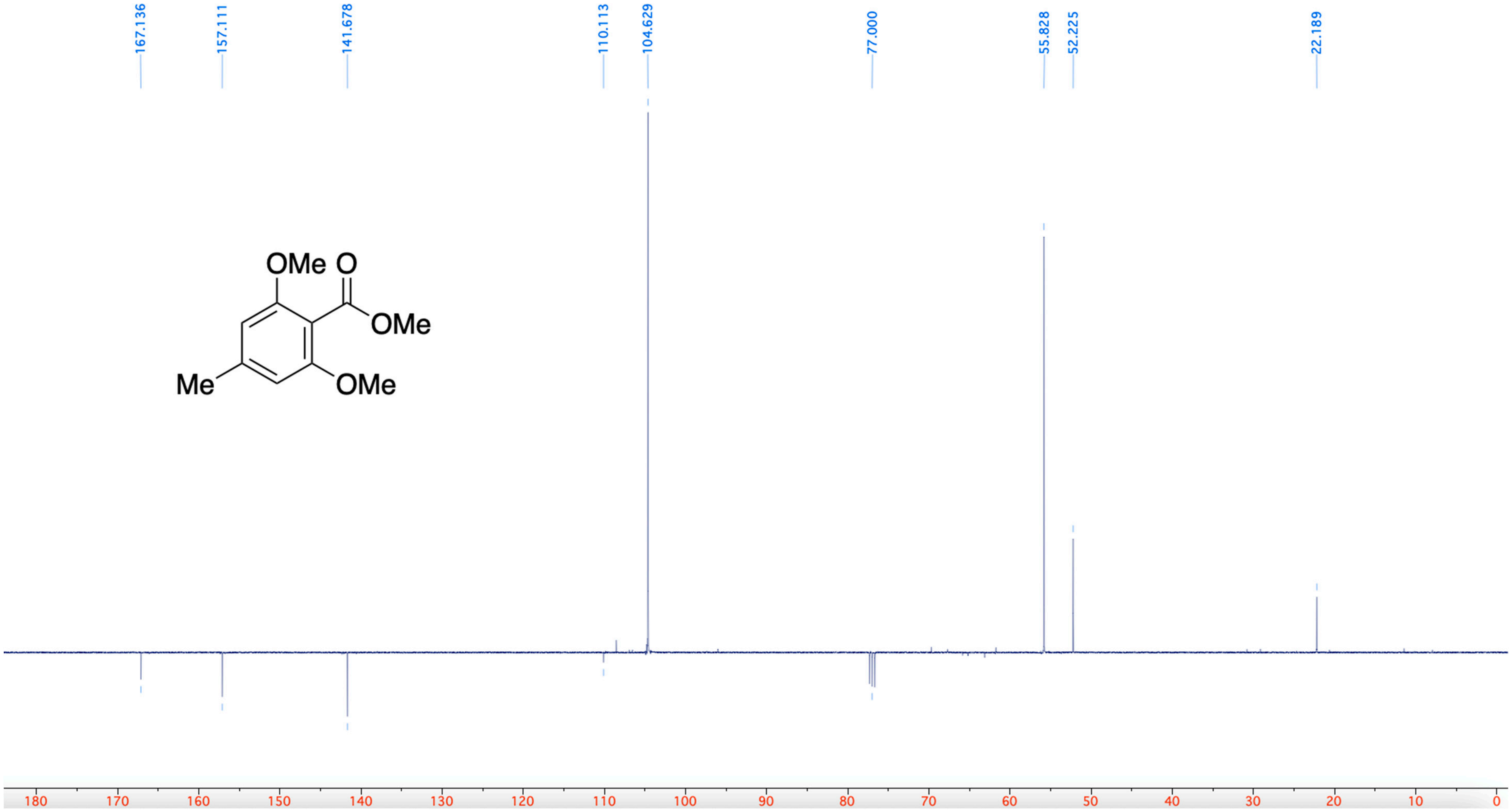

Figure S12: 100 MHz ^{13}C NMR spectrum of **19** ($CDCl_3$)

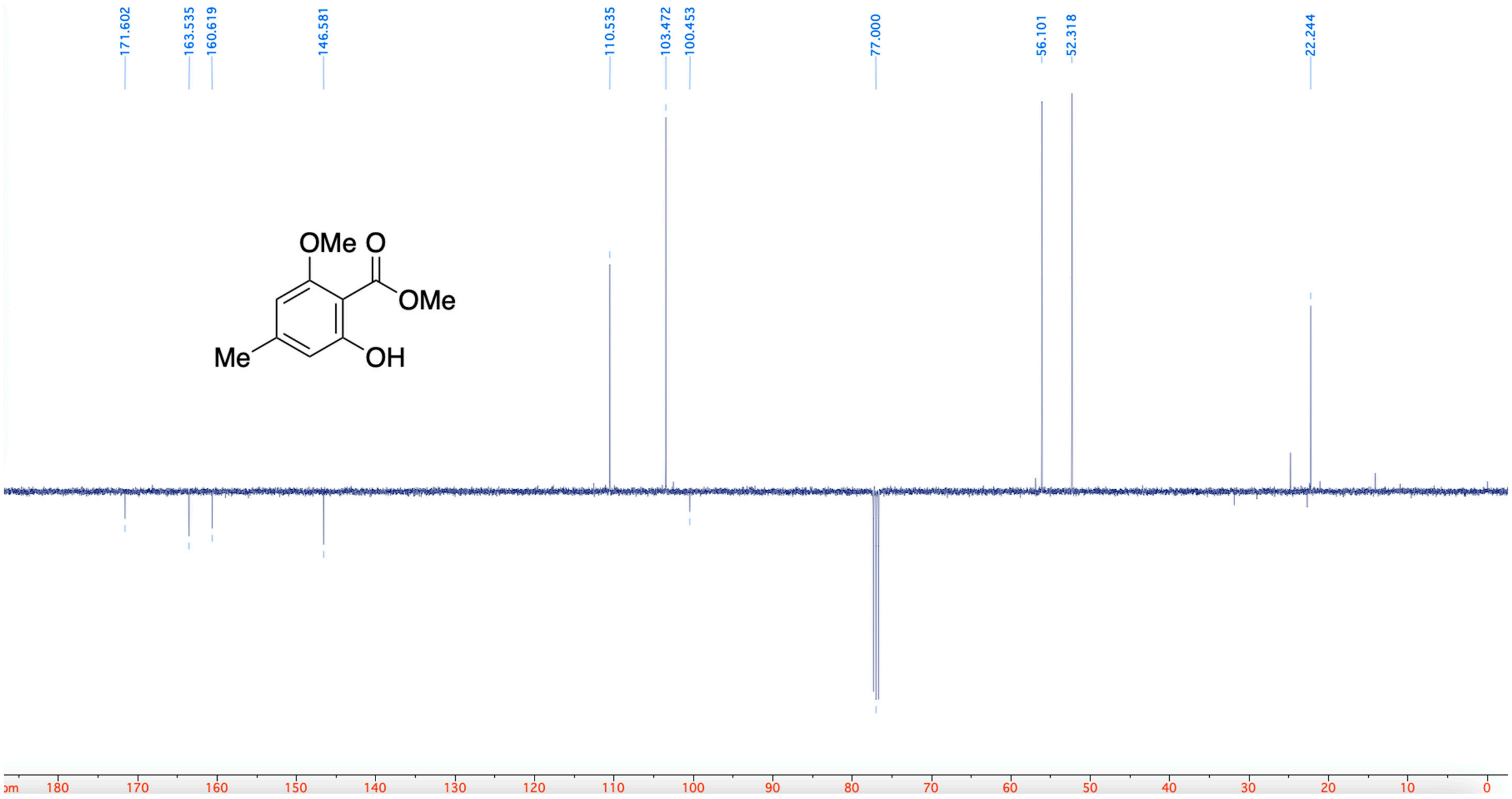

Figure S13: 400 MHz ^{1}H NMR spectrum of **20** ($CDCl_3$)

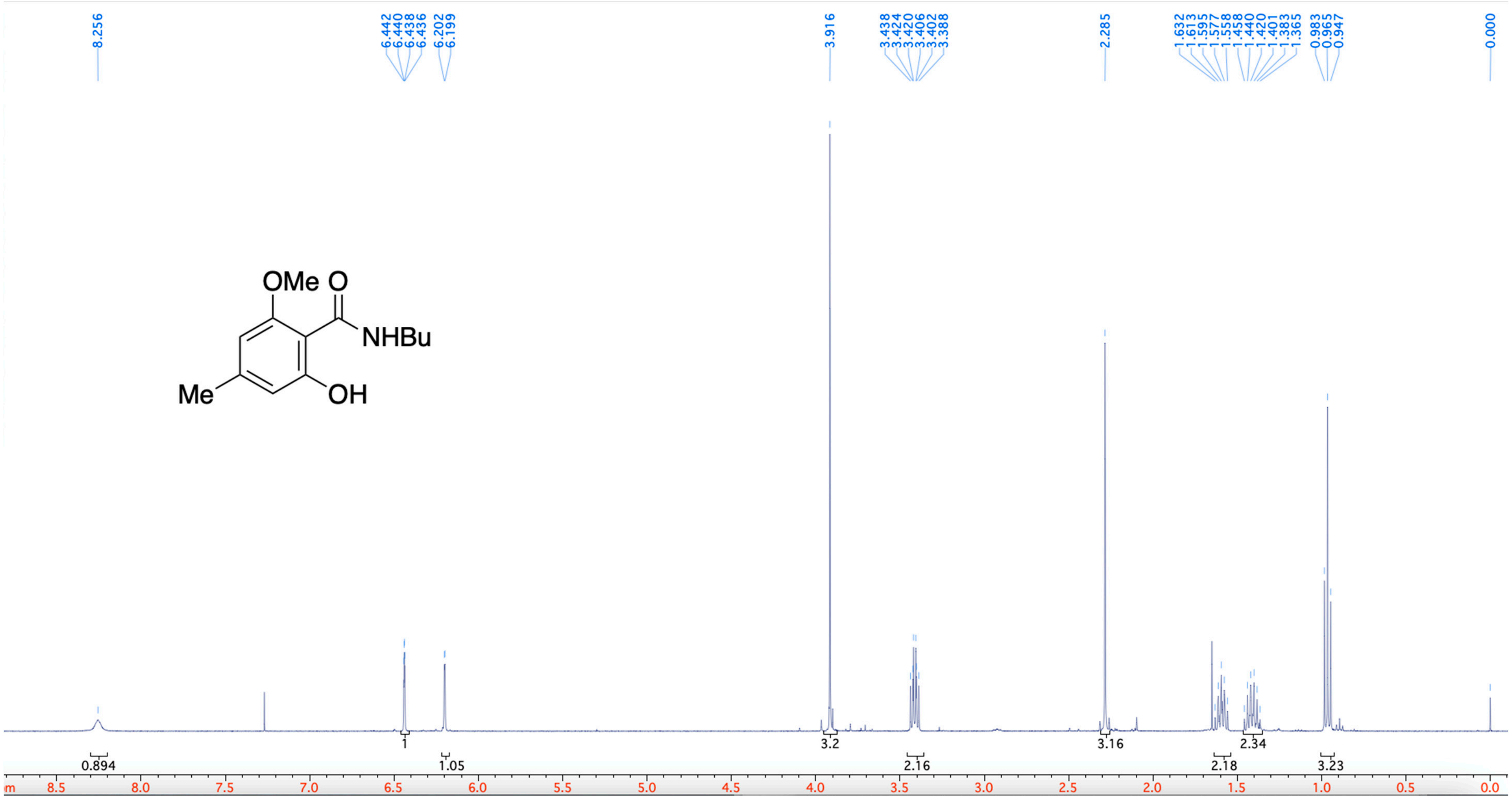

Figure S14: 100 MHz ^{13}C NMR spectrum of **20** ($CDCl_3$)

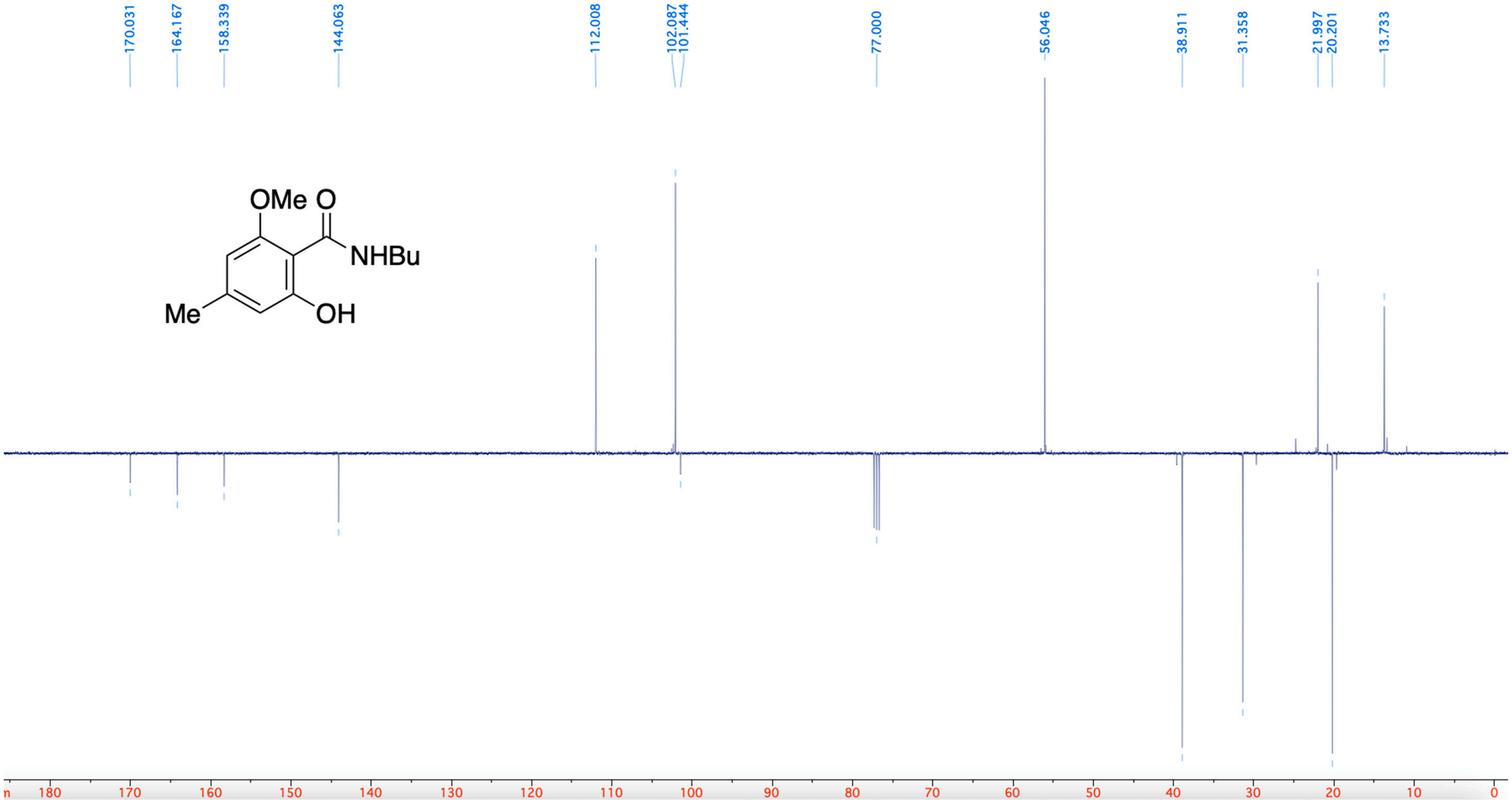

Figure S15: 400 MHz ^{1}H NMR spectrum of **22** ($CDCl_3$)

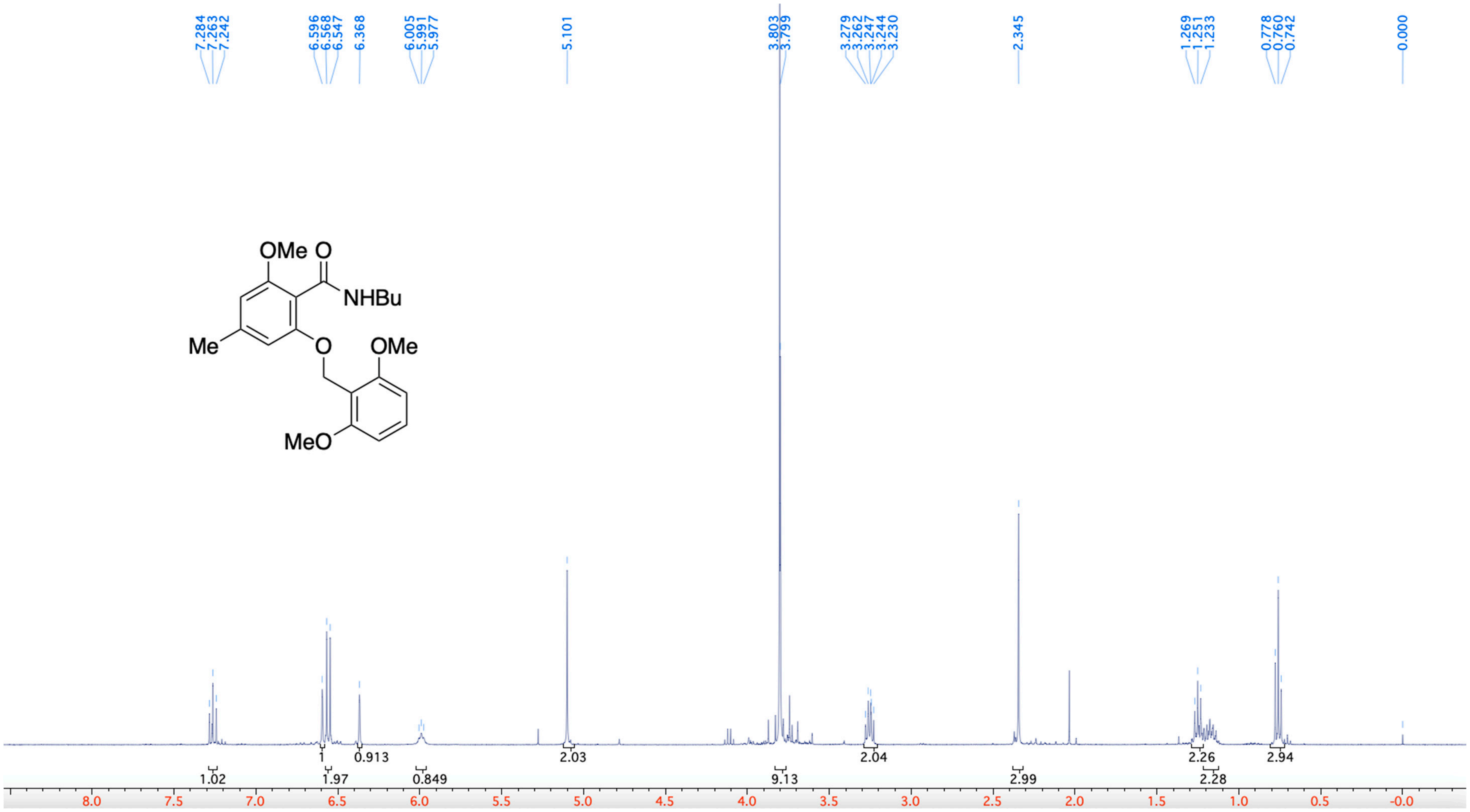

Figure S16: 100 MHz ^{13}C NMR spectrum of **22** ($CDCl_3$)

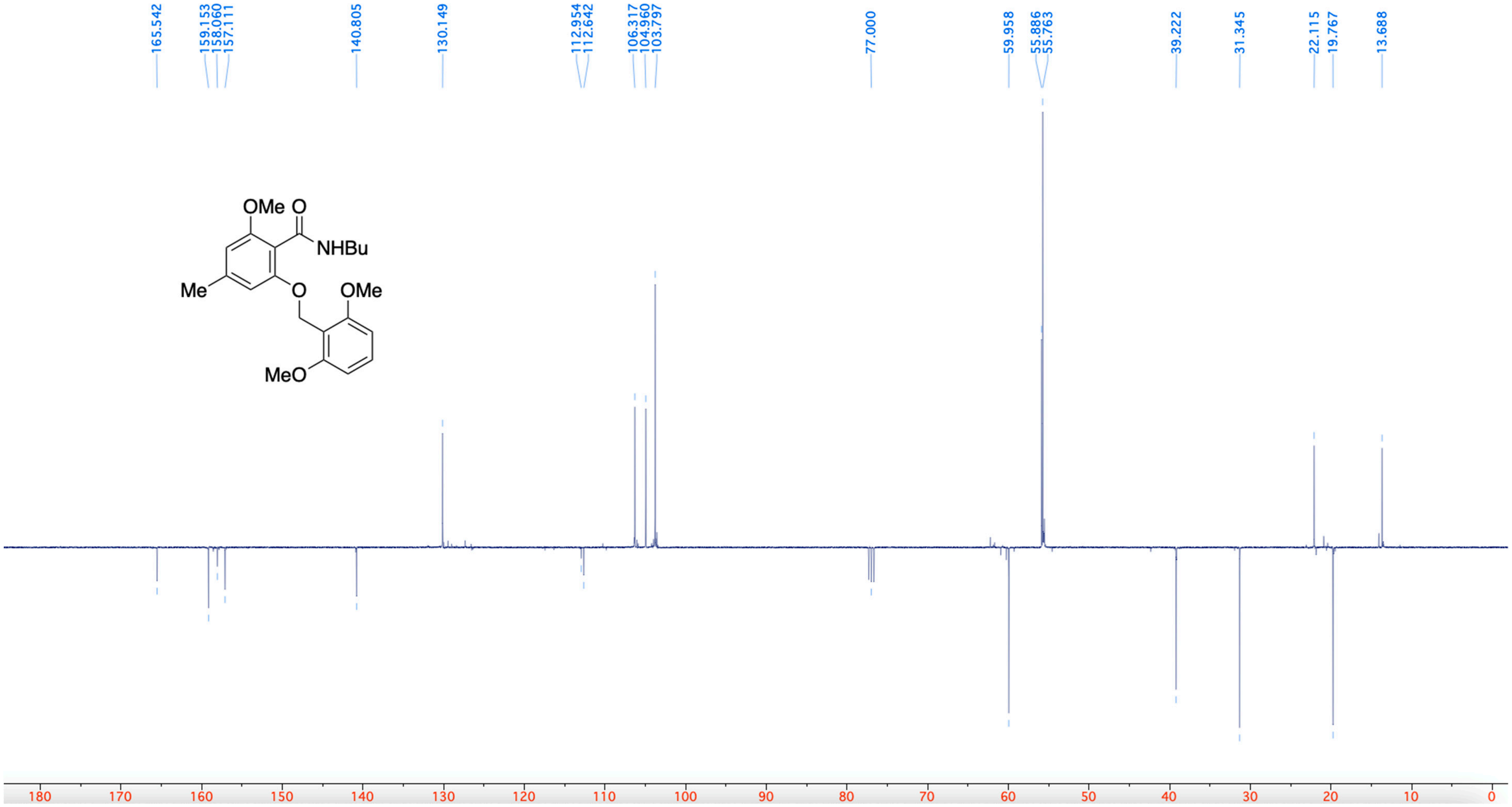

Figure S17: 300 MHz ^{1}H NMR spectrum of **30** ($CDCl_3$)

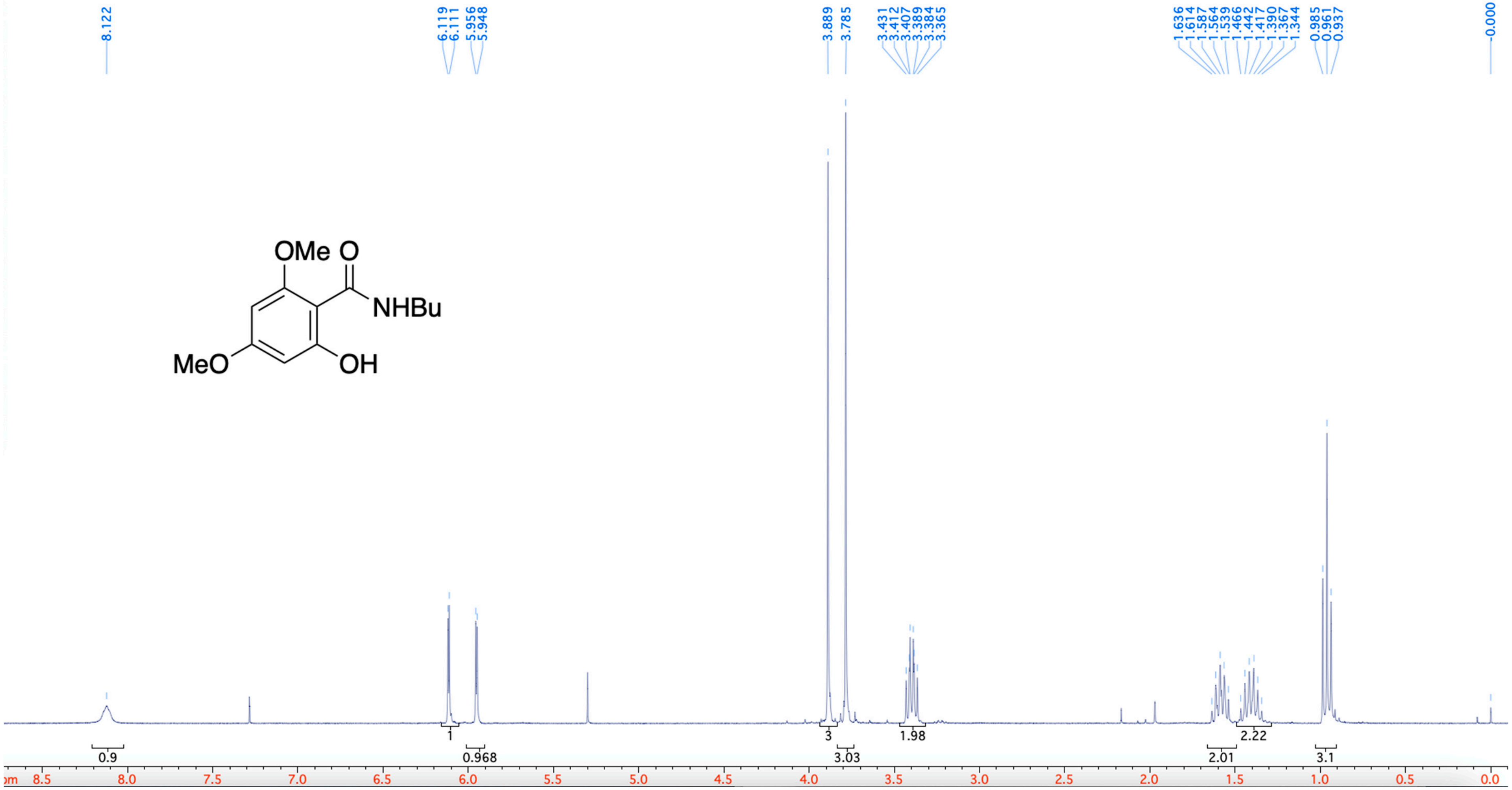

Figure S18: 75 MHz ^{13}C NMR spectrum of **30** ($CDCl_3$)

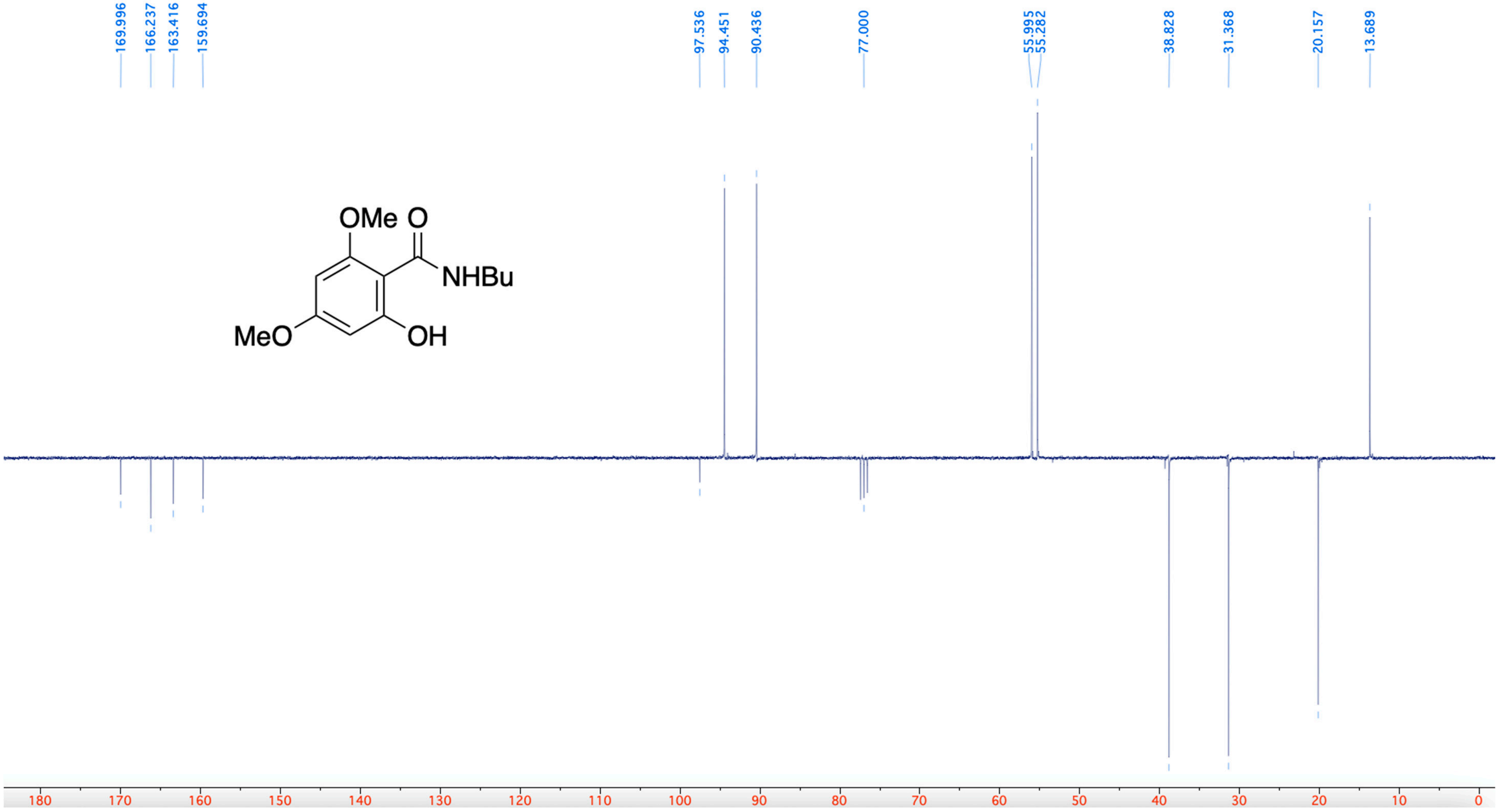

Figure S19: 400 MHz ^{1}H NMR spectrum of **34** ($CDCl_3$)

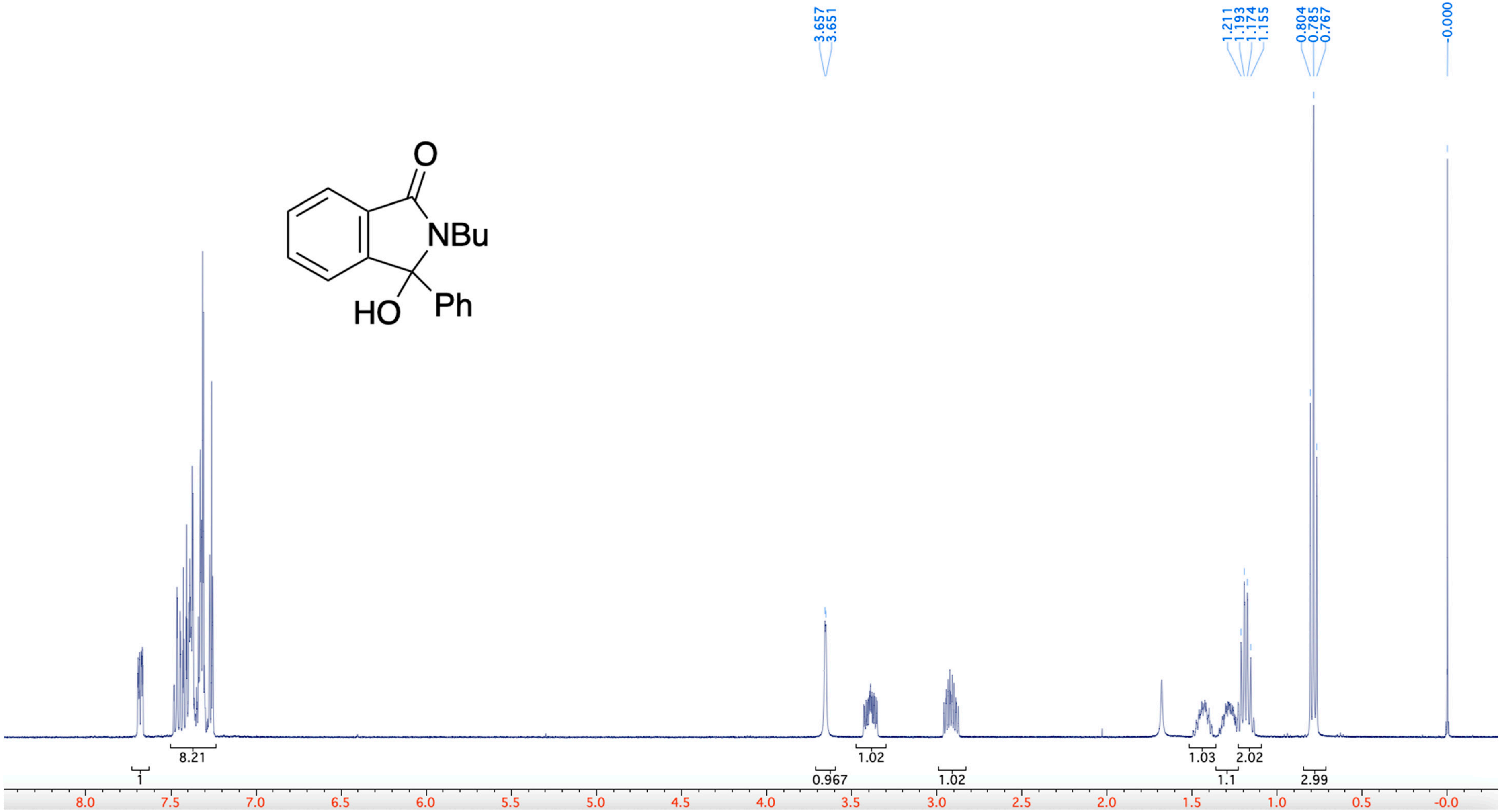

Figure S20: 75 MHz ^{13}C NMR spectrum of **34** ($CDCl_3$)

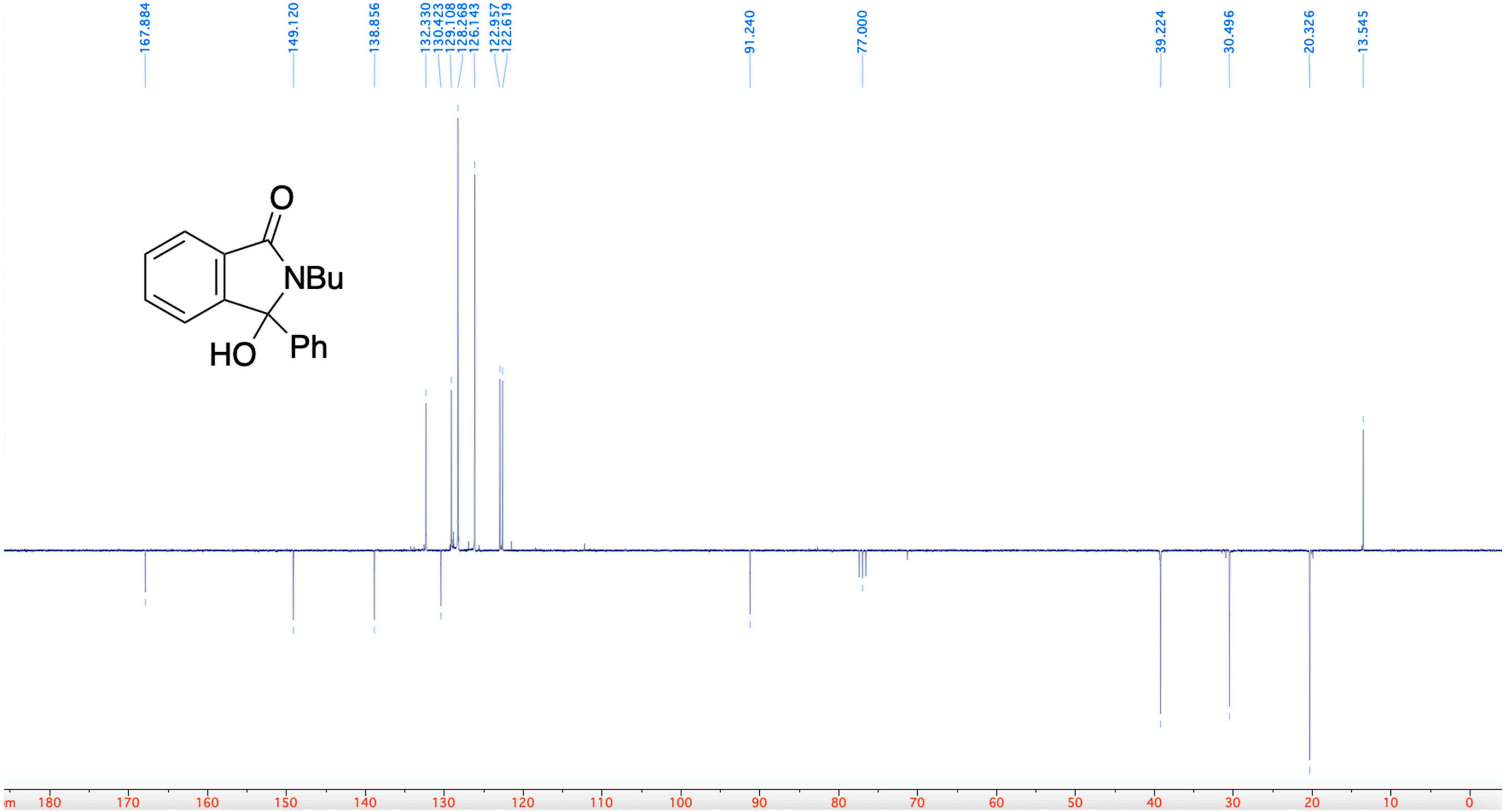

Figure S21: 500 MHz ^{1}H NMR spectrum of **39** ($CDCl_3$)

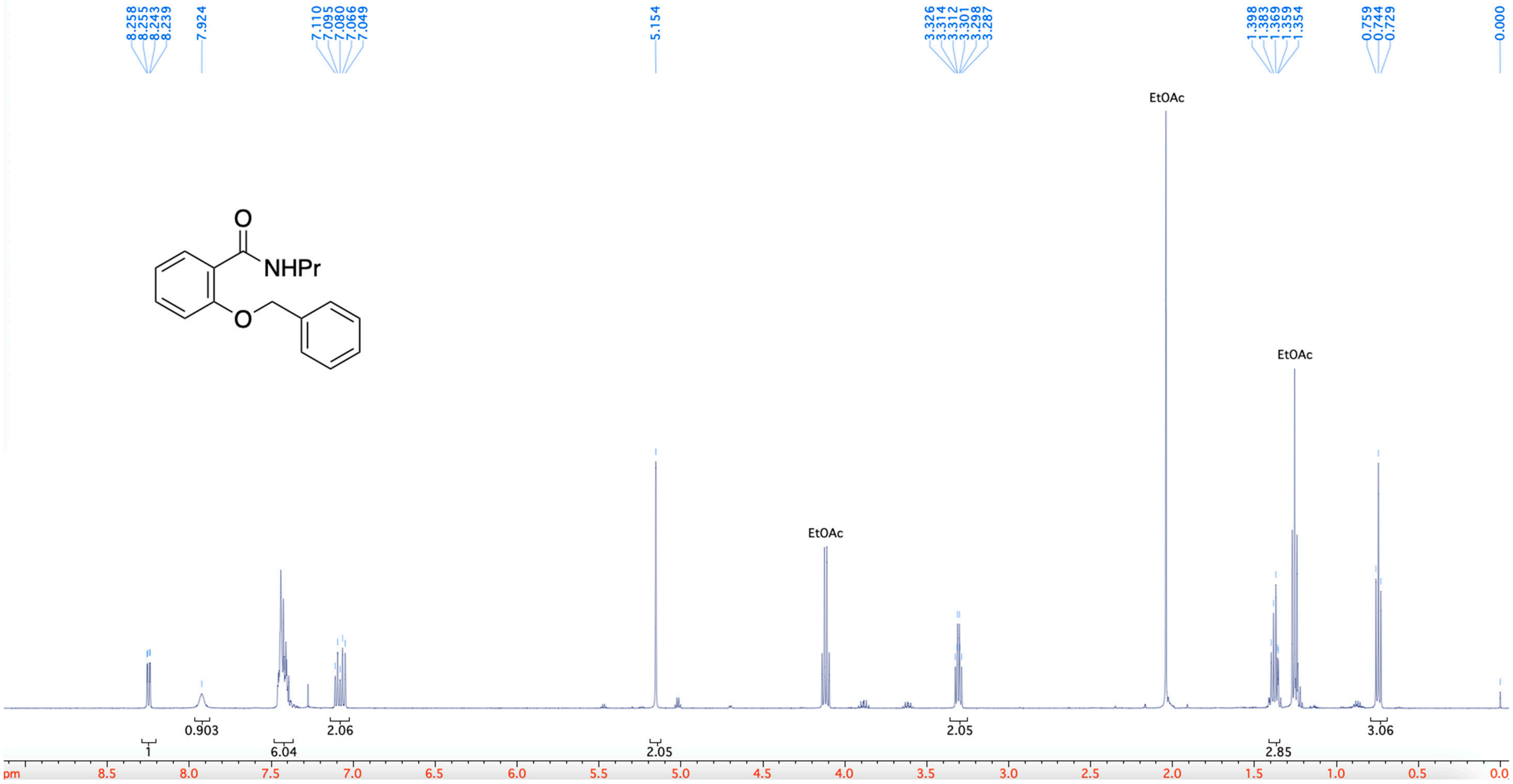

Figure S22: 100 MHz ^{13}C NMR spectrum of **39** ($CDCl_3$)

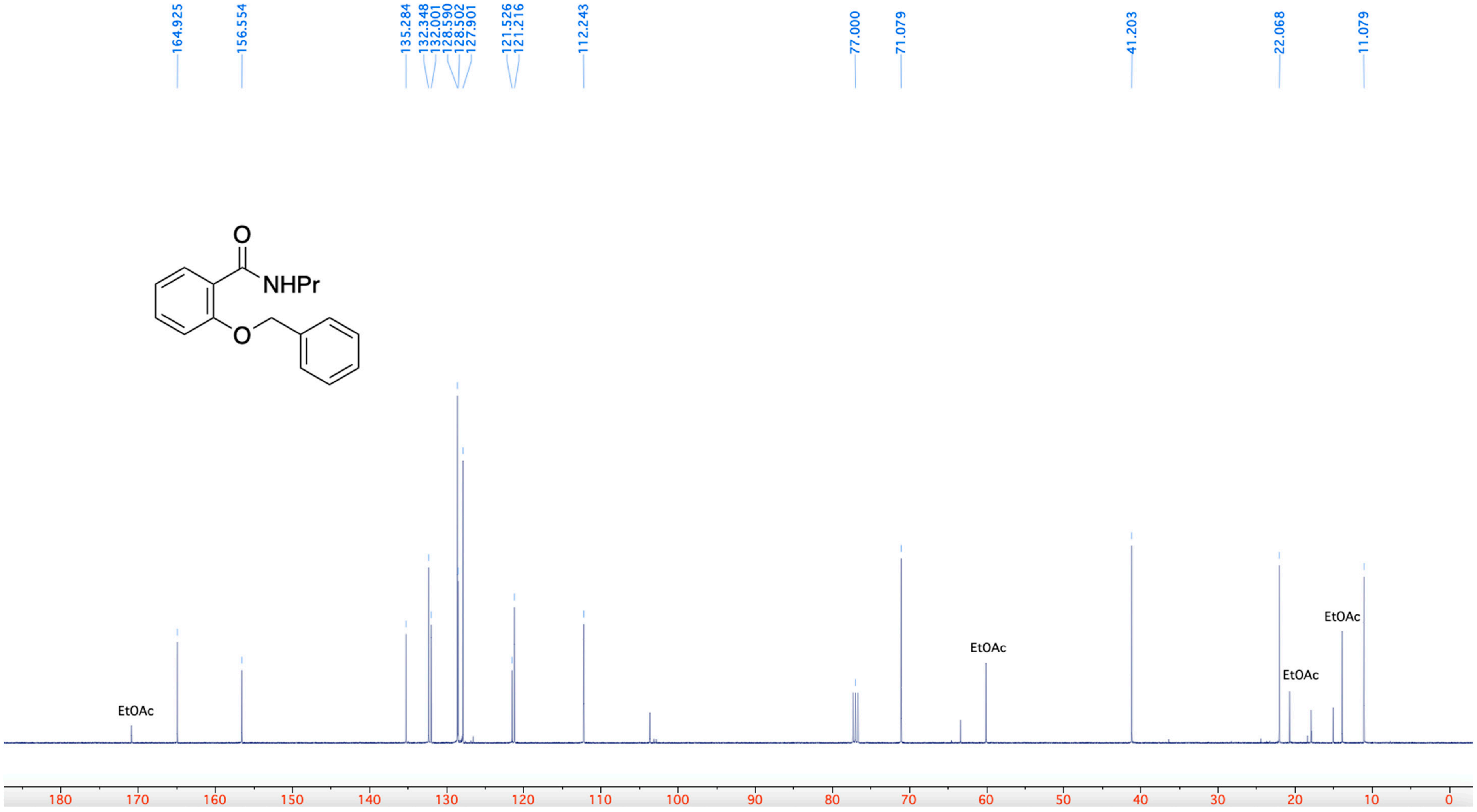

Figure S23: 400 MHz ^{1}H NMR spectrum of **40** ($CDCl_3$)

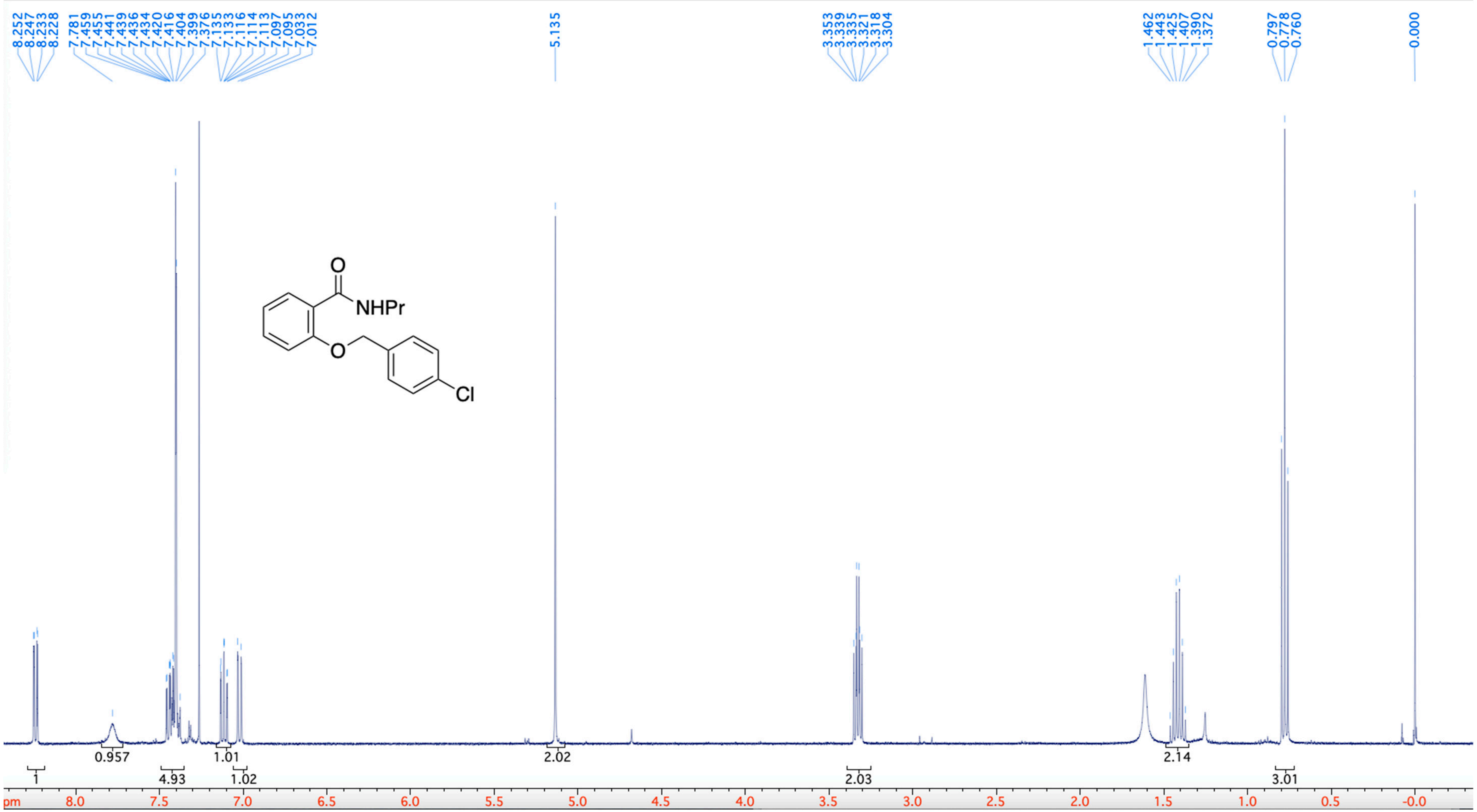

Figure S24: 100 MHz ^{13}C NMR spectrum of **40** ($CDCl_3$)

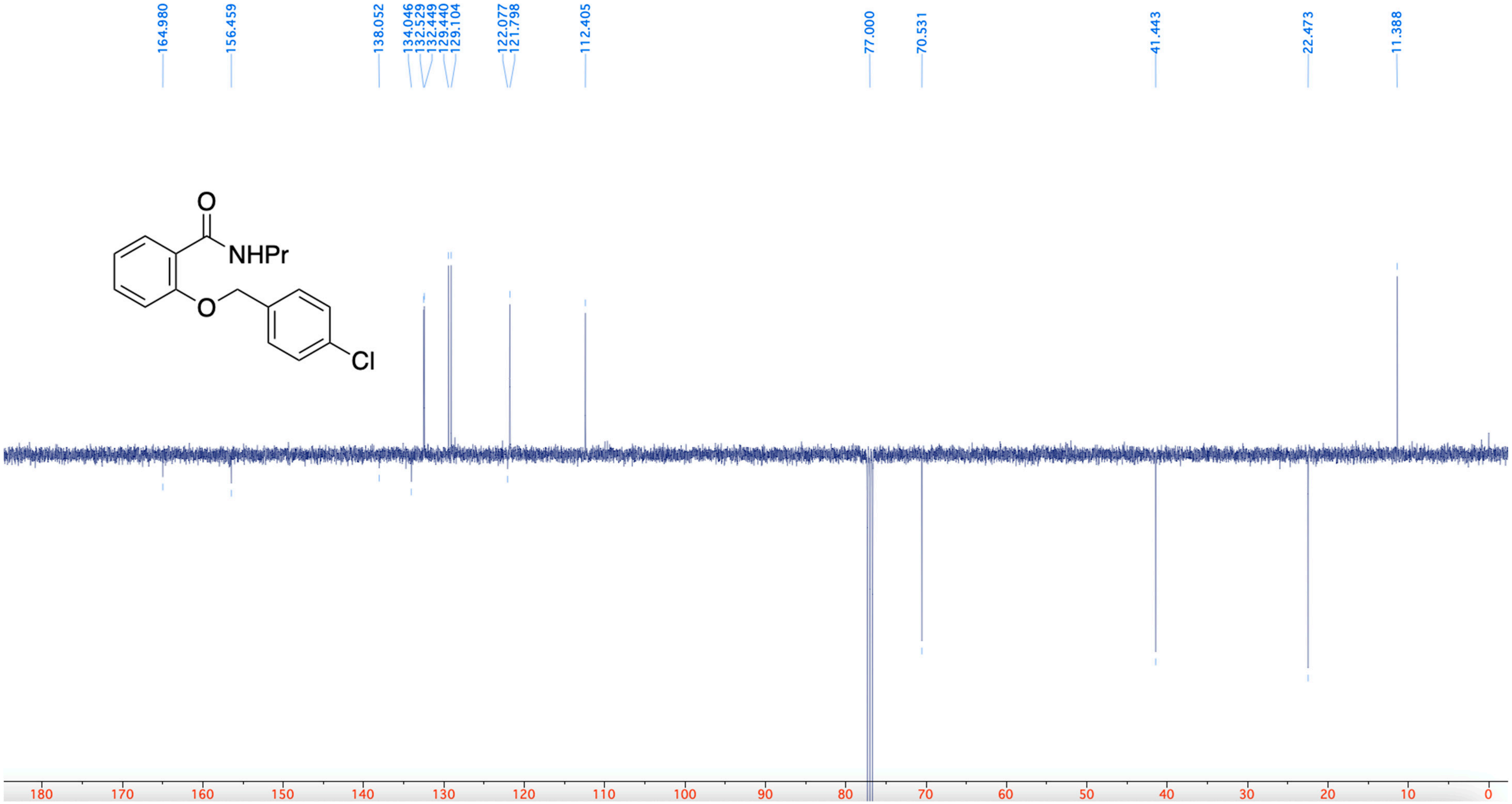

Figure S25: 400 MHz ^{1}H NMR spectrum of **41** ($CDCl_3$)

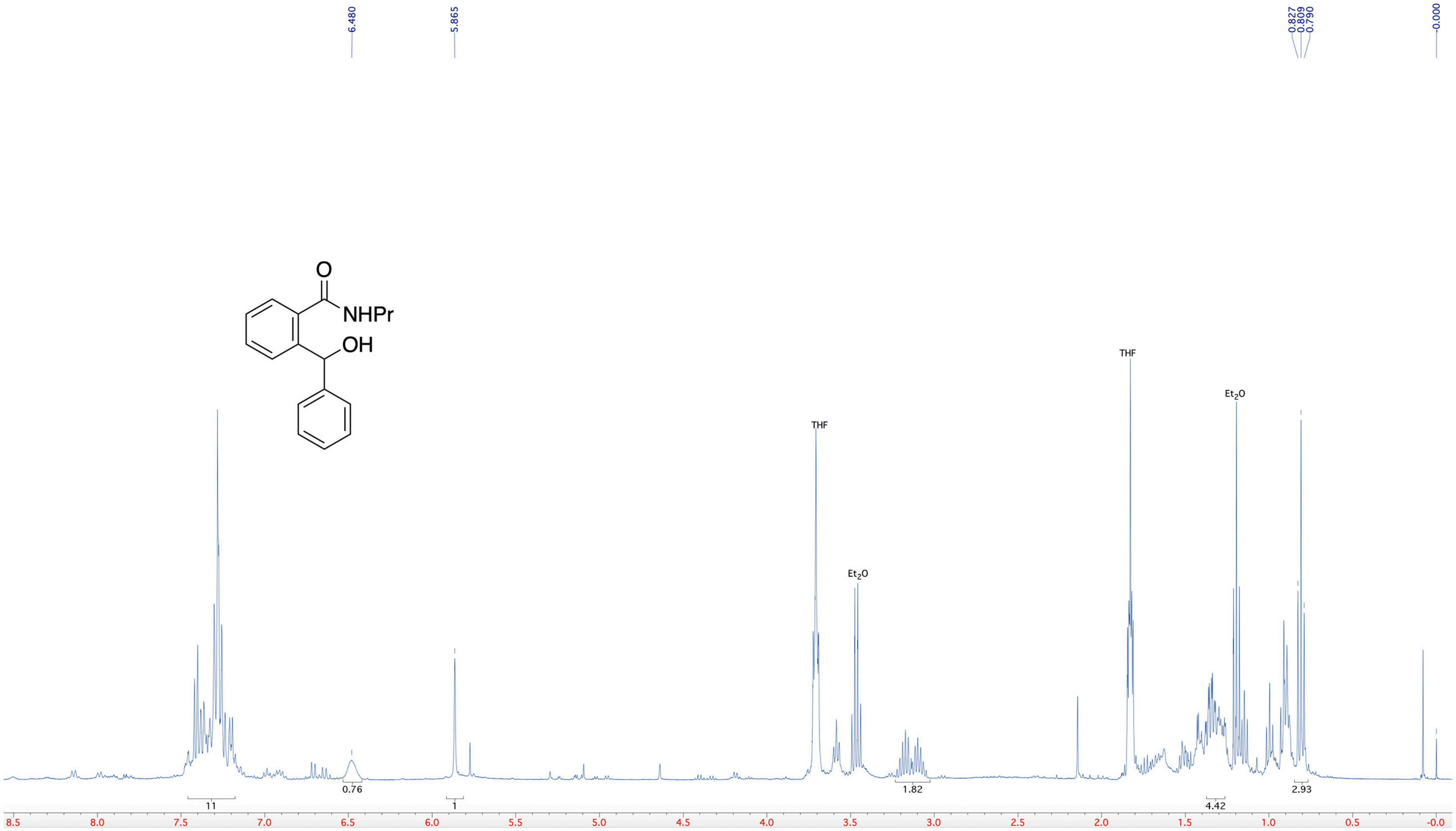

Figure S26: 100 MHz ^{13}C NMR spectrum of **41** ($CDCl_3$)

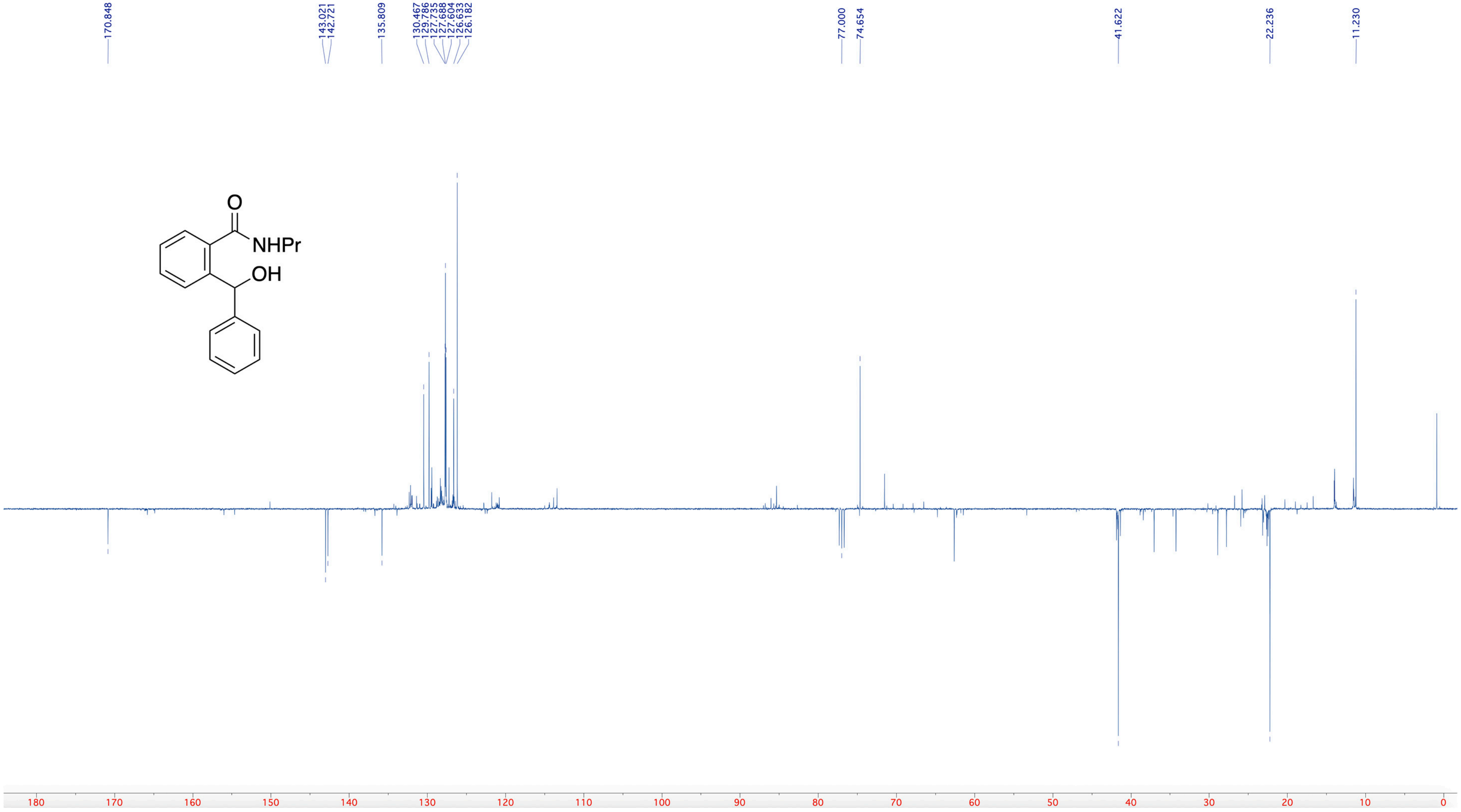

Figure S27: 400 MHz ^{1}H NMR spectrum of **42** ($CDCl_3$)

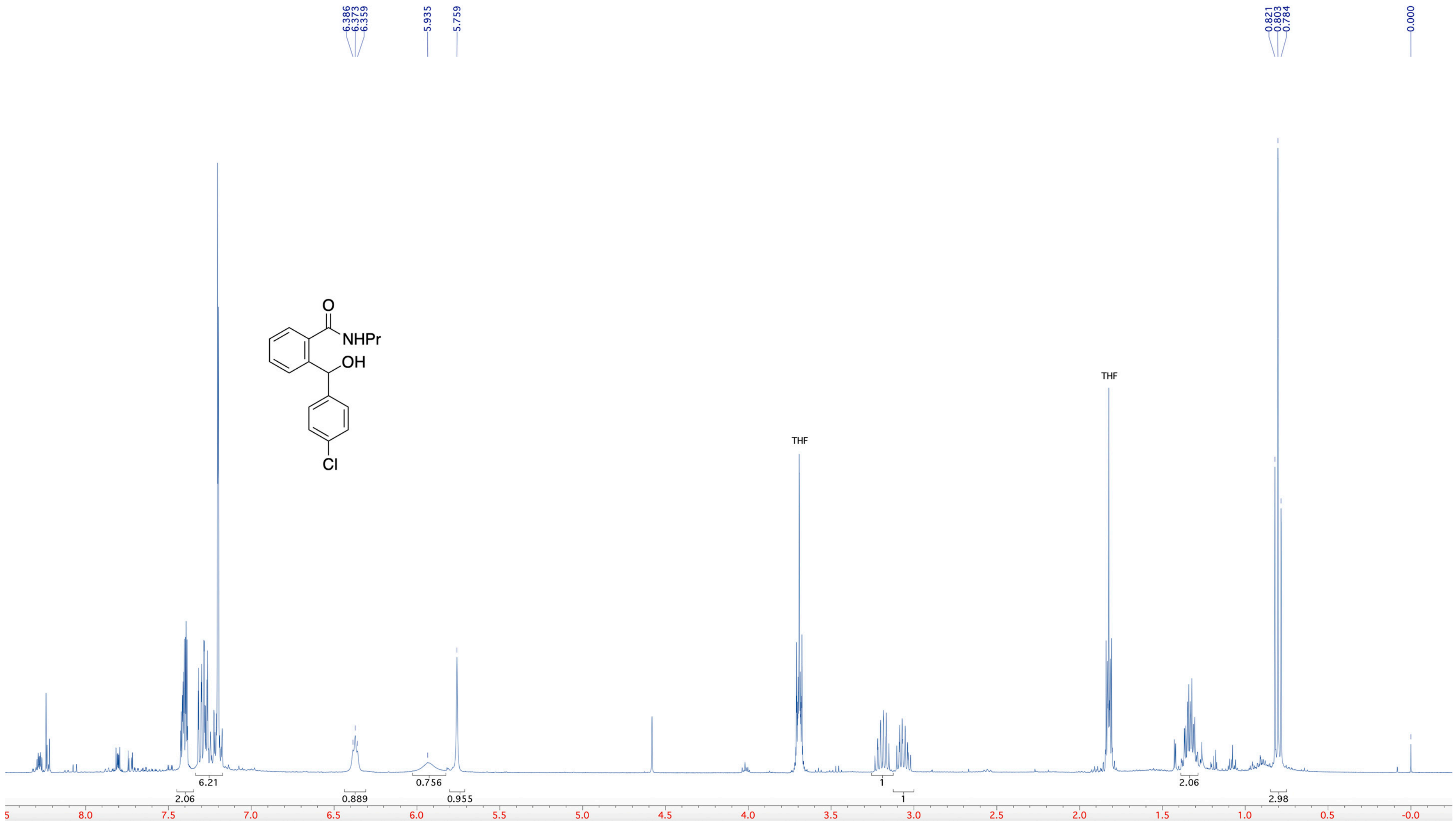

Figure S28: 100 MHz ^{13}C NMR spectrum of **42** ($CDCl_3$)

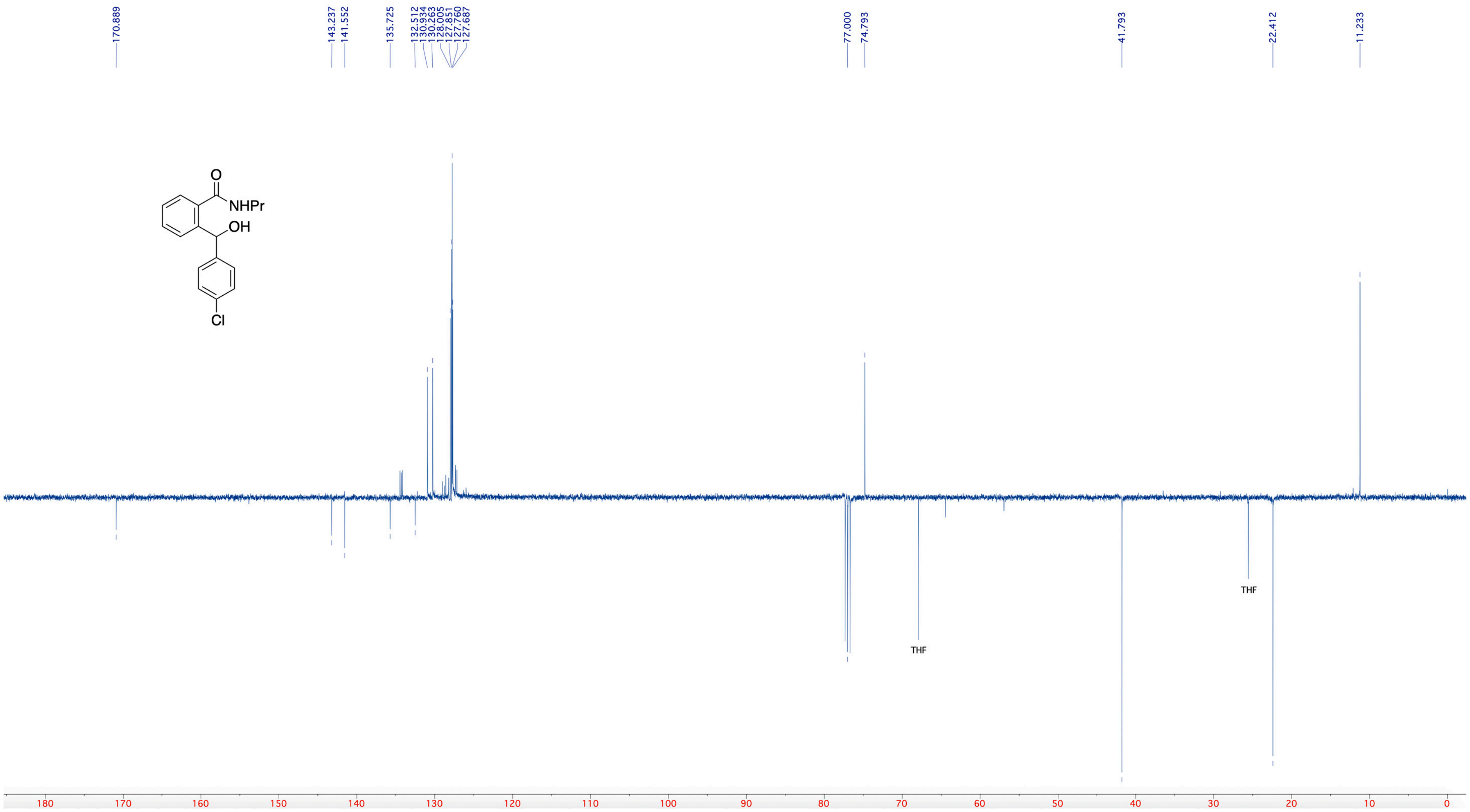

Figure S29: 500 MHz ^{1}H NMR spectrum of **43** ($CDCl_3$)

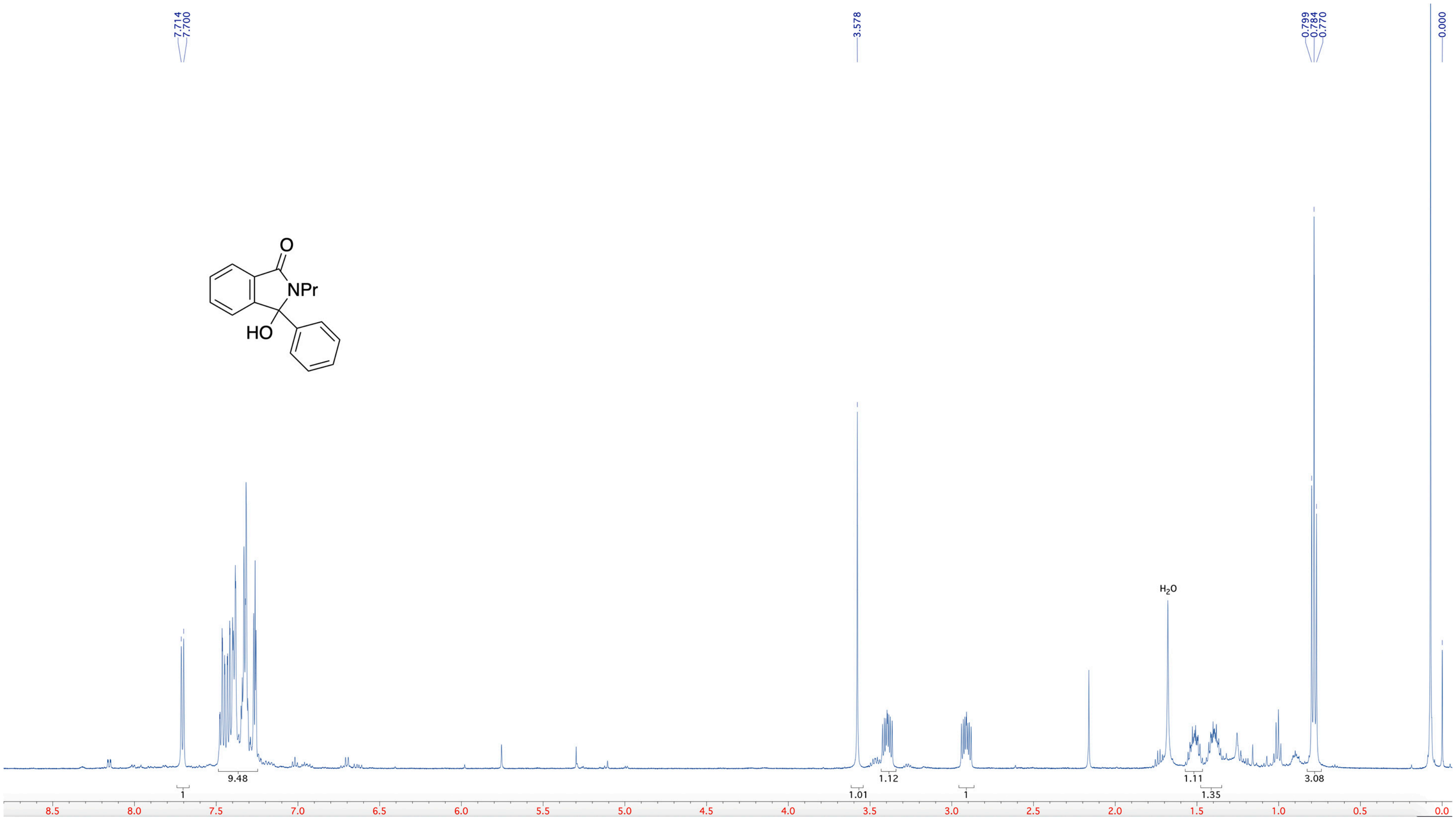

Figure S30: 100 MHz ^{13}C NMR spectrum of **43** ($CDCl_3$)

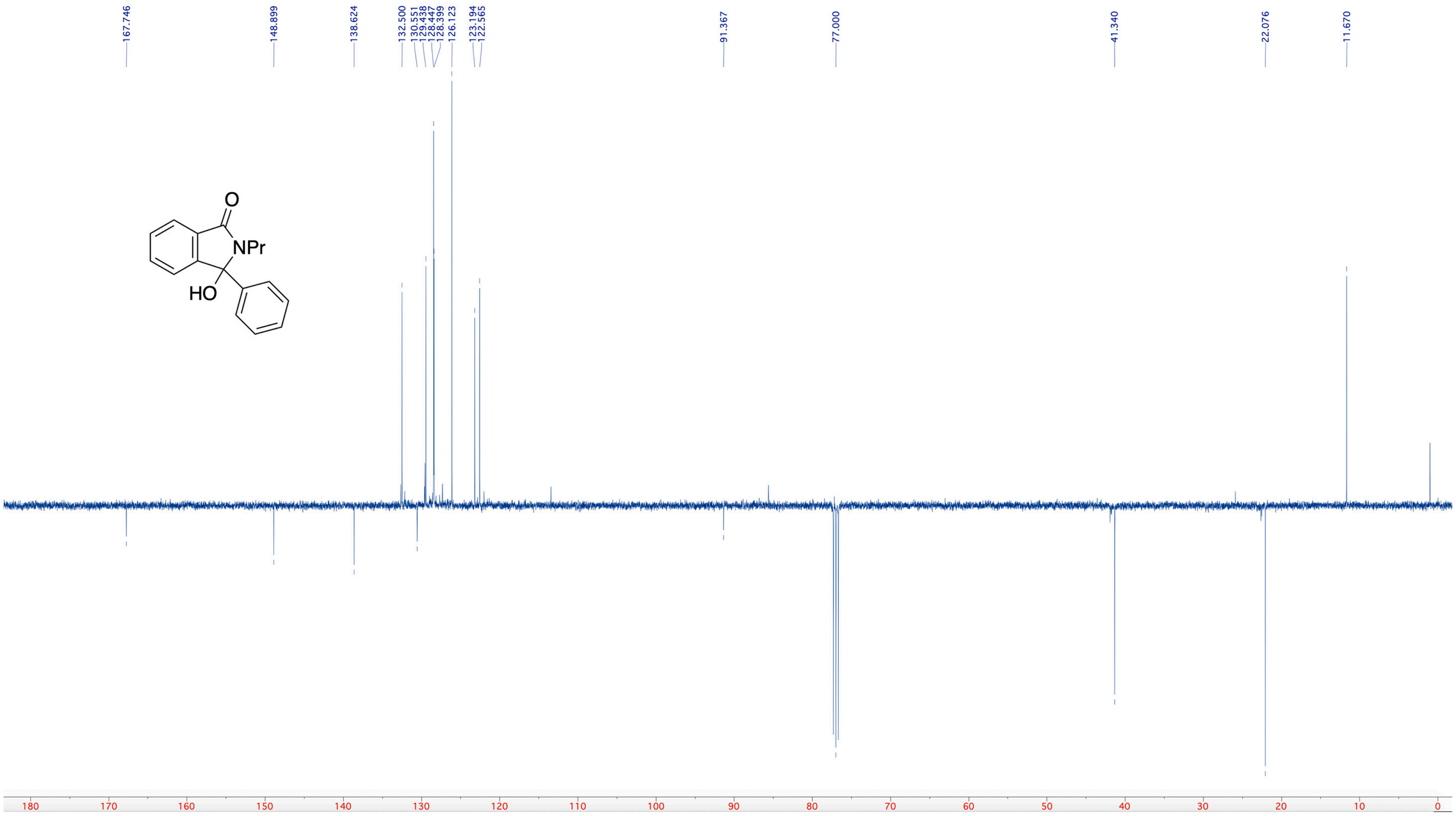

Figure S31: 400 MHz ^{1}H NMR spectrum of **44** ($CDCl_3$)

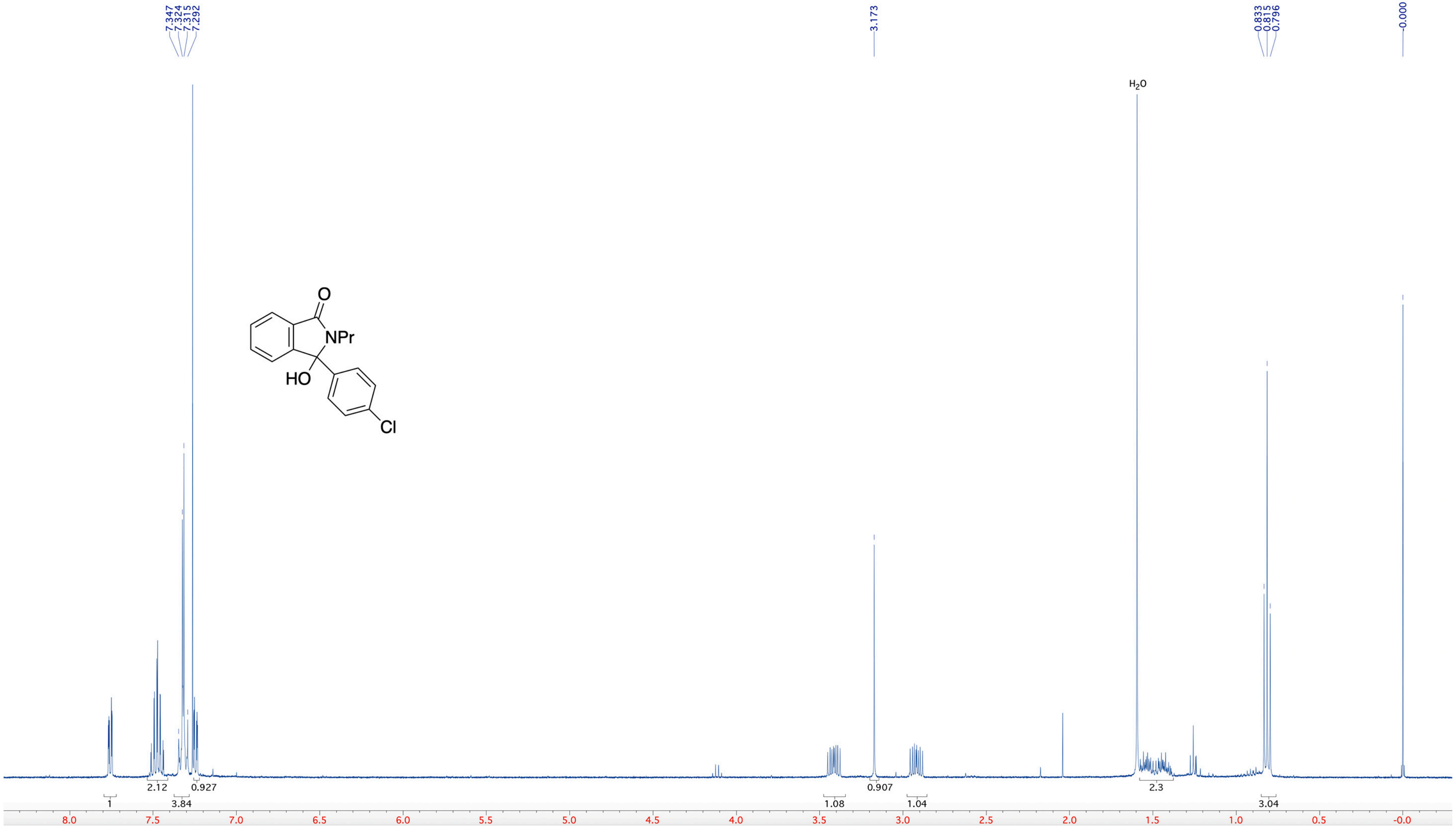

Figure S32: 100 MHz ^{13}C NMR spectrum of **44** ($CDCl_3$)

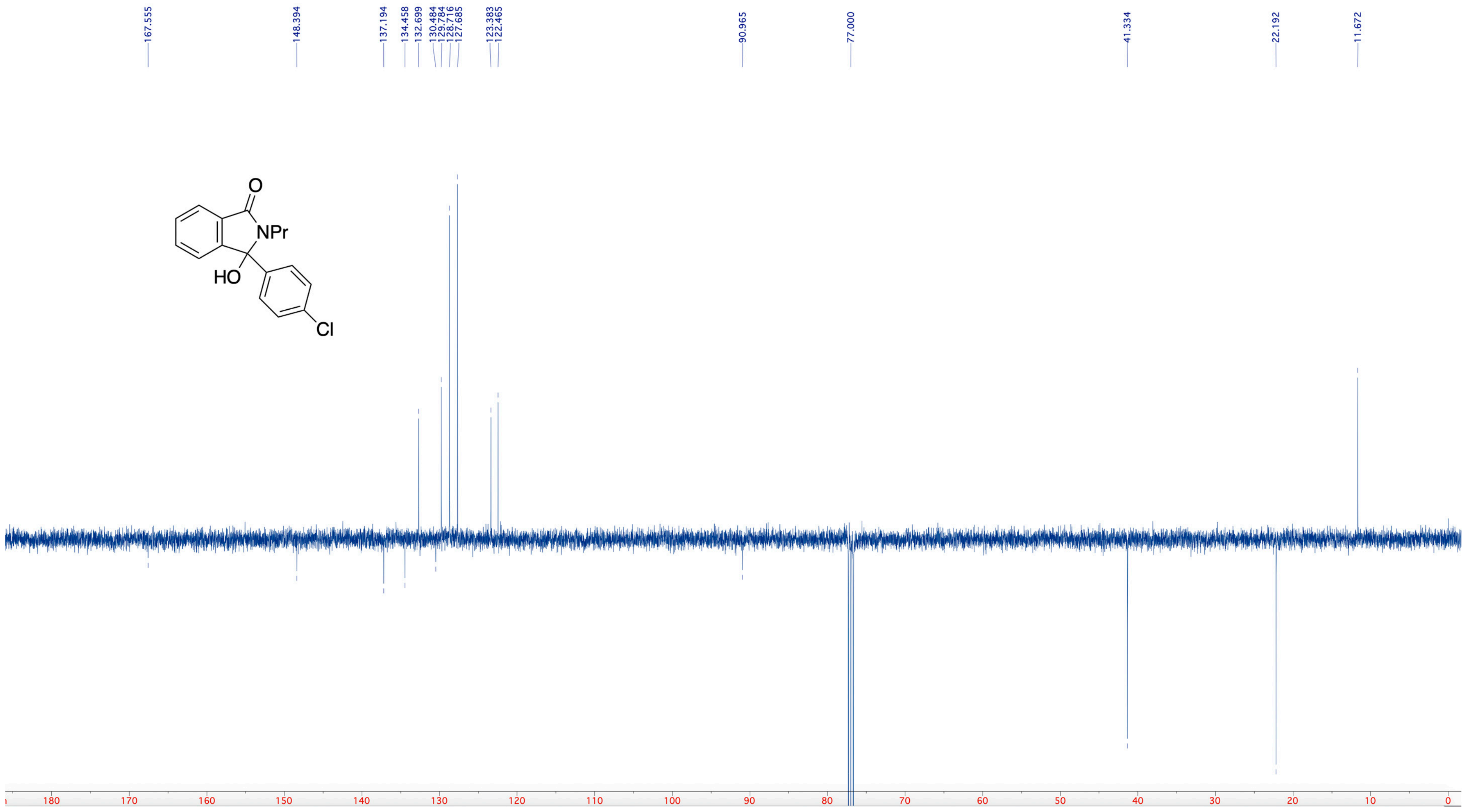

Figure S33: 400 MHz ^{1}H NMR spectrum of **35** ($CDCl_3$)

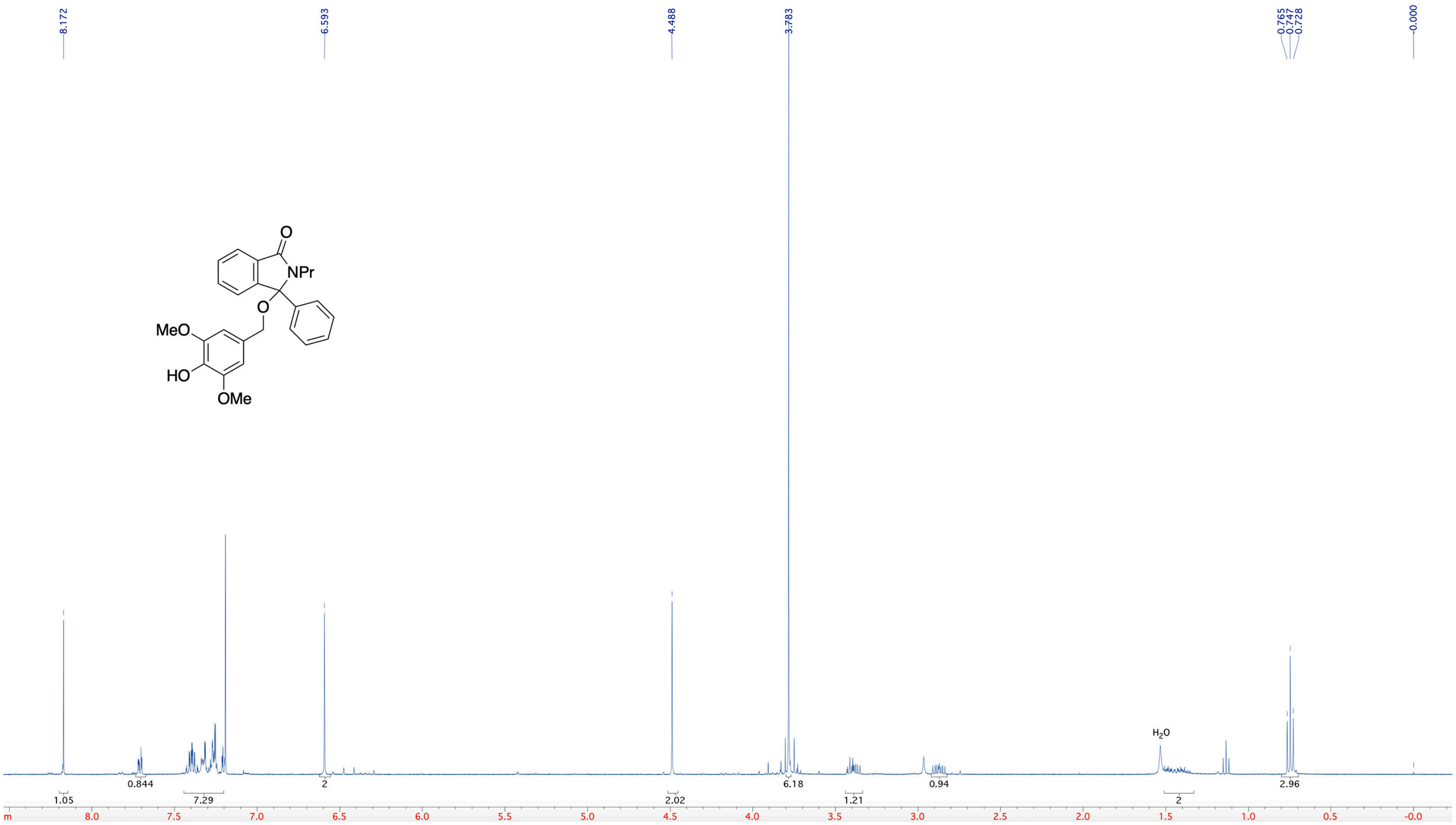

Figure S34: 100 MHz ^{13}C NMR spectrum of **35** ($CDCl_3$)

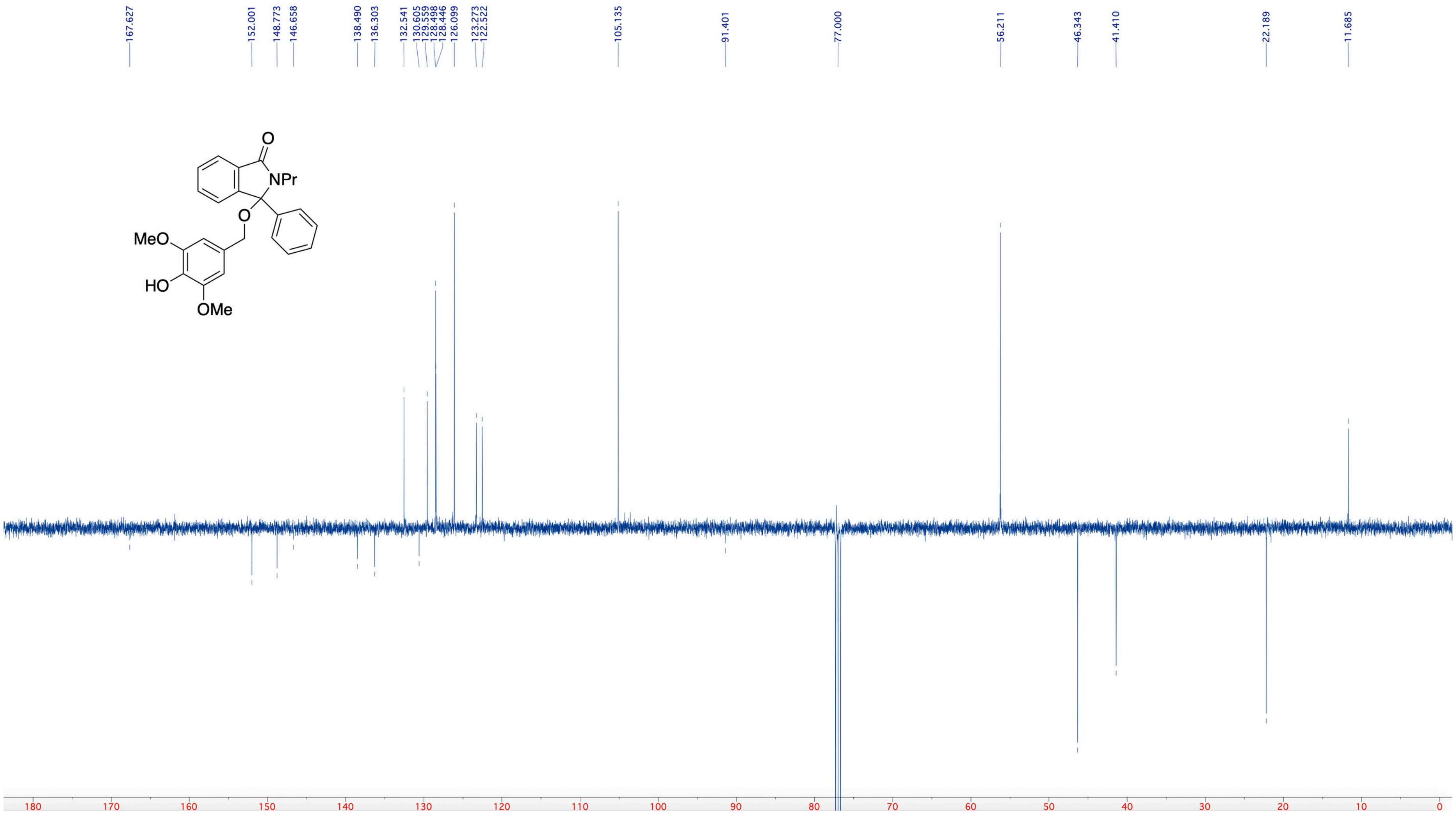

Figure S35: 400 MHz ^{1}H NMR spectrum of **36** ($CDCl_3$)

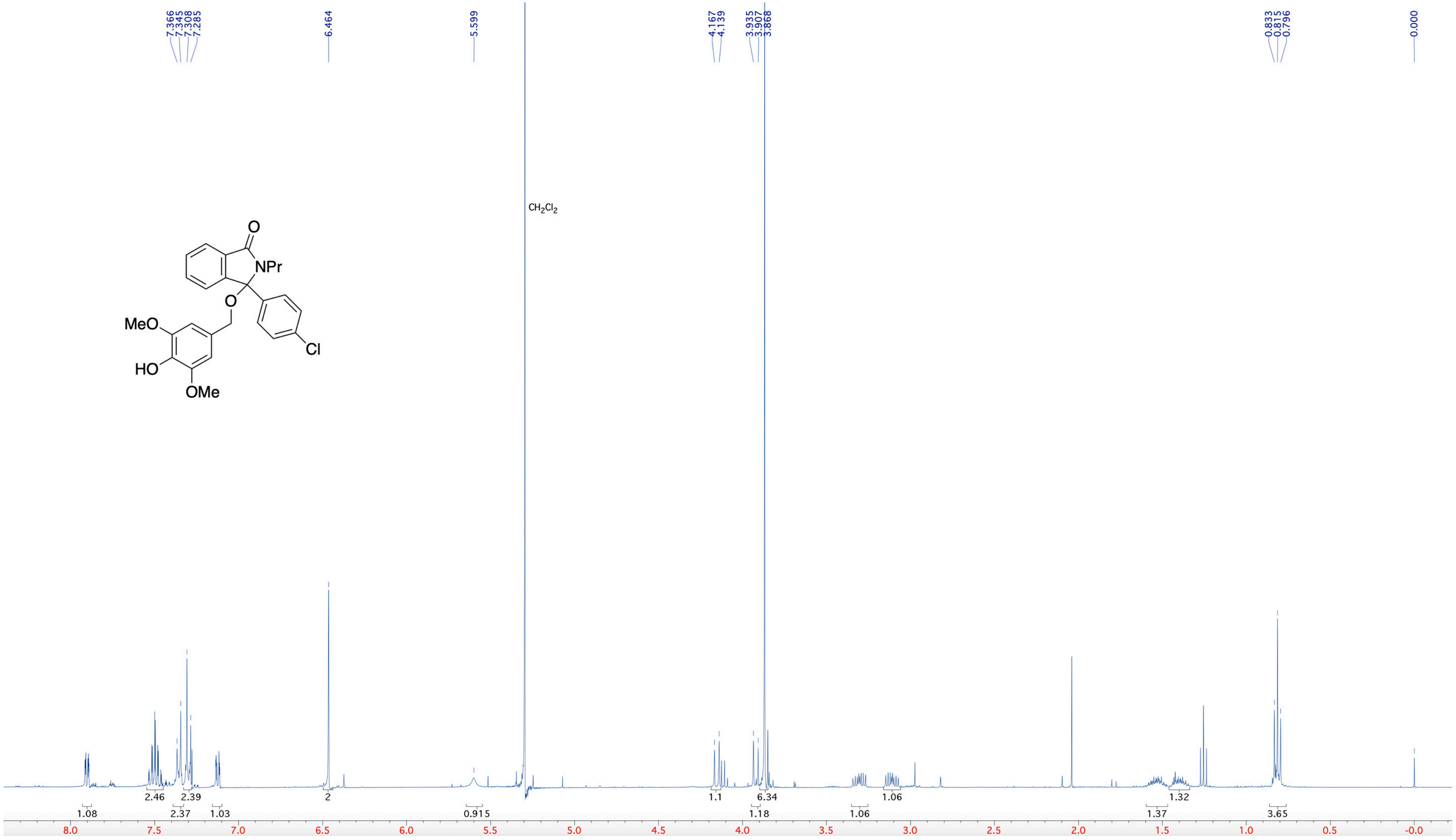

Figure S36: 100 MHz ^{13}C NMR spectrum of **36** ($CDCl_3$)

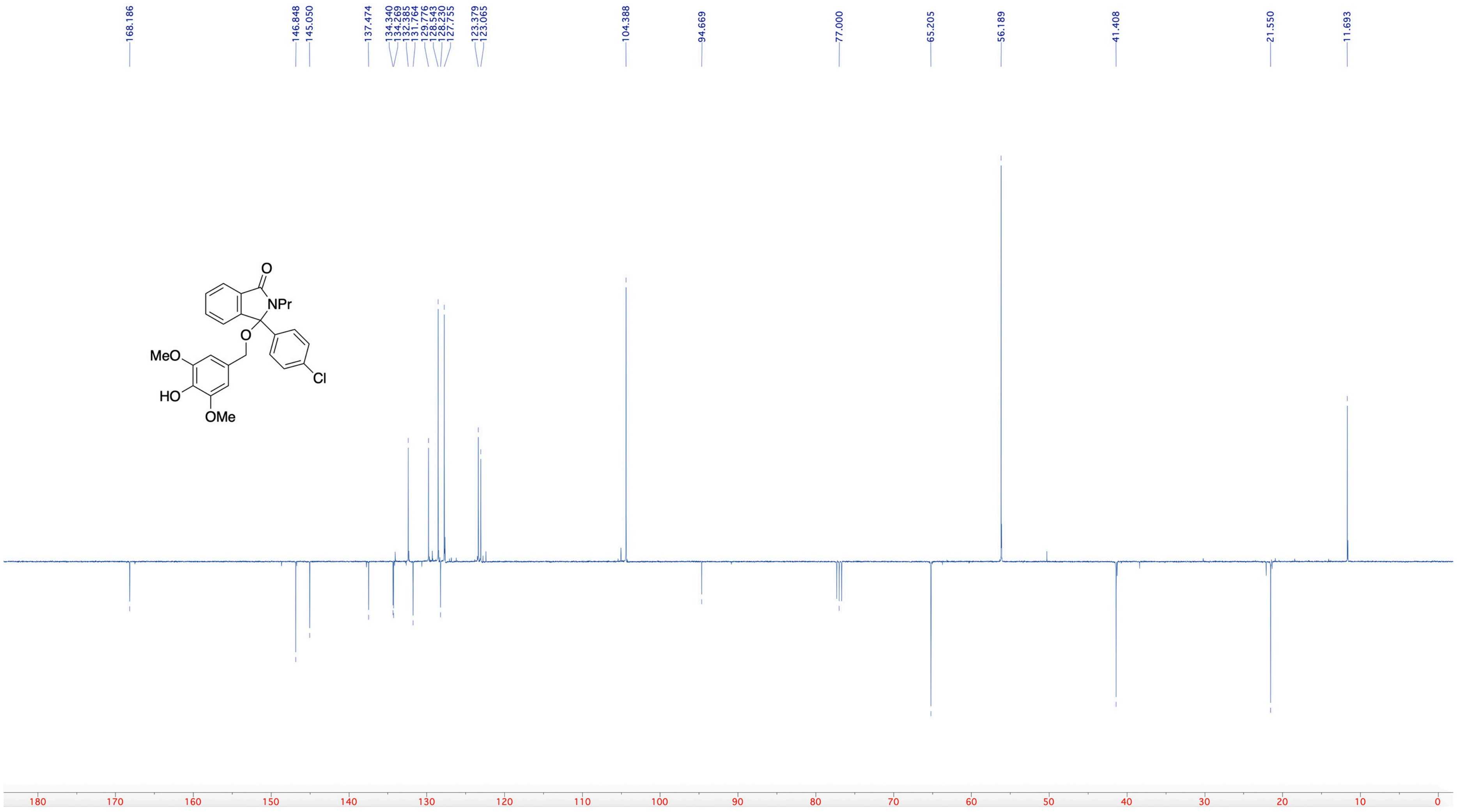

Supplement: Supplementary file 1 [file molecules-29-04722-s001.zip › molecules-3227084-supplementary.pdf]
